# The NASA Roadmap to Ocean Worlds

**DOI:** 10.1089/ast.2018.1955

**Published:** 2018-12-29

**Authors:** Amanda R. Hendrix, Terry A. Hurford, Laura M. Barge, Michael T. Bland, Jeff S. Bowman, William Brinckerhoff, Bonnie J. Buratti, Morgan L. Cable, Julie Castillo-Rogez, Geoffrey C. Collins, Serina Diniega, Christopher R. German, Alexander G. Hayes, Tori Hoehler, Sona Hosseini, Carly J.A. Howett, Alfred S. McEwen, Catherine D. Neish, Marc Neveu, Tom A. Nordheim, G. Wesley Patterson, D. Alex Patthoff, Cynthia Phillips, Alyssa Rhoden, Britney E. Schmidt, Kelsi N. Singer, Jason M. Soderblom, Steven D. Vance

**Affiliations:** ^1^ Planetary Science Institute, Tucson, Arizona.; ^2^ NASA Goddard Space Flight Center, Greenbelt, Maryland.; ^3^ Jet Propulsion Laboratory, California Institute of Technology, Pasadena, California.; ^4^ Astrogeology Science Center, U.S. Geological Survey, Flagstaff, Arizona.; ^5^ Scripps Institution of Oceanography, La Jolla, California.; ^6^ Physics and Astronomy Department, Wheaton College, Norton, Massachusetts.; ^7^ Woods Hole Oceanographic Institution, Woods Hole, Massachusetts.; ^8^ Cornell Center for Astrophysics and Planetary Science, Cornell University, Ithaca, New York.; ^9^ NASA Ames Research Center, Mountain View, California.; ^10^ Southwest Research Institute, Boulder, Colorado.; ^11^ Lunar and Planetary Laboratory, University of Arizona, Tucson, Arizona.; ^12^ Department of Earth Sciences, The University of Western Ontario, London, Ontario, Canada.; ^13^ NASA HQ/Universities Space Association, Washington, District of Columbia.; ^14^ Applied Physics Laboratory, Johns Hopkins University, Laurel, Maryland.; ^15^ School of Earth & Atmospheric Sciences, Georgia Institute of Technology, Atlanta, Georgia.; ^16^ Department of Earth, Atmospheric and Planetary Sciences, Massachusetts Institute of Technology, Cambridge, Massachusetts.

**Keywords:** Roadmap, Enceladus, Titan, Europa, Triton, NASA.

## Abstract

In this article, we summarize the work of the NASA Outer Planets Assessment Group (OPAG) Roadmaps to Ocean Worlds (ROW) group. The aim of this group is to assemble the scientific framework that will guide the exploration of ocean worlds, and to identify and prioritize science objectives for ocean worlds over the next several decades. The overarching goal of an Ocean Worlds exploration program as defined by ROW is to “identify ocean worlds, characterize their oceans, evaluate their habitability, search for life, and ultimately understand any life we find.” The ROW team supports the creation of an exploration program that studies the full spectrum of ocean worlds, that is, not just the exploration of known ocean worlds such as Europa but candidate ocean worlds such as Triton as well. The ROW team finds that the confirmed ocean worlds Enceladus, Titan, and Europa are the highest priority bodies to target in the near term to address ROW goals. Triton is the highest priority candidate ocean world to target in the near term. A major finding of this study is that, to map out a coherent Ocean Worlds Program, significant input is required from studies here on Earth; rigorous Research and Analysis studies are called for to enable some future ocean worlds missions to be thoughtfully planned and undertaken. A second finding is that progress needs to be made in the area of collaborations between Earth ocean scientists and extraterrestrial ocean scientists.

## 1. Introduction

The 2016 Congressional Commerce, Justice, Science, and Related Agencies Appropriations Bill (hereafter CJS) directed NASA to create an Ocean Worlds Exploration program, using a mix of programs already established within NASA. Their direction for this program was to seek out and discover extant life in habitable worlds (HW) in the Solar System. In support of these efforts, the Outer Planets Assessment Group (OPAG), directed by NASA's Planetary Science Division (PSD), formed the Roadmaps to Ocean Worlds (ROW) group to assemble the scientific framework guiding the exploration of ocean worlds. ROW was given the following charter:

- Identify and prioritize science objectives for ocean worlds over the next several decades
- Design roadmap(s) to explore these worlds to address science objectives (including mission sequences, considering a sustained exploration effort)
- Assess where each ocean world fits into the overall roadmap
- Summarize broad mission concepts (considering mission dependencies and international cooperation)
- Recommend technology development and detailed mission studies in support of the next Decadal Survey

The ROW team consists of 70–80 Earth ocean and planetary science community experts, from NASA centers, academia, and private research institutions. Inputs for this article were assembled through teleconferences and face-to-face meetings, usually in conjunction with OPAG meetings. Townhalls were held at the Lunar and Planetary Science Conference (LPSC) and Astrobiology Science Conference (AbSciCon), and updates were given at OPAG and Small Bodies Assessment Group (SBAG) meetings to inform the wider community of ROW progress and to gather further inputs.

This article describes the scientific content and priorities for investigations that are needed for the exploration of ocean worlds. Such investigations would be carried out by a robotic flight program that would measure needed quantities at ocean worlds, and by research efforts to characterize important physical processes potentially at work on ocean worlds.

## 2. Confirmed and Candidate Ocean Worlds

For the purposes of this article, and to bound the extent of a future Ocean Worlds Program, we define an “ocean world” as a body with a current liquid ocean (not necessarily global). All bodies in our Solar System that plausibly can have, or are known to have, an ocean will be considered in this framework. The Earth is a well-studied ocean world that we use as a reference (“ground truth”) and point of comparison. We do not include the ice giant planets as ocean worlds.

There are several—if not many—ocean worlds or potential ocean worlds in our Solar System, all targets for future NASA missions in the quest for understanding the distribution of life in the Solar System. In considering ocean worlds, there are several with confirmed oceans, several candidates that exhibit hints of possible oceans, and worlds in our Solar System that may theoretically harbor oceans but about which not enough is currently known to determine whether an ocean exists. As a philosophy, it is critical to consider all of these worlds to understand the origin and development of oceans and life in different worlds: Does life originate and take hold in some ocean worlds and not others, and why? Thus, the ROW team supports the creation of a program that studies the full spectrum of ocean worlds; if only one or two ocean worlds are explored and life is discovered (or not), we will not fully understand the distribution of life, its origin and variability, and the repeatability of its occurrences in the Solar System.

The House CJS Appropriations 2016 bill explicitly identifies Europa, Enceladus, and Titan as ocean worlds. We have considered that Enceladus, Europa, Titan, Ganymede, and Callisto have known subsurface oceans, as determined from geophysical measurements by the *Galileo* and *Cassini* spacecraft. These are confirmed/known ocean worlds. Europa and Enceladus stand out as ocean worlds with evidence for communication between the ocean and the surface, as well as the potential for interactions between the oceans and a rocky seafloor, which is important for habitability considerations. The subsurface oceans of Titan, Ganymede, and Callisto are expected to be covered by relatively thick ice shells, making exchange processes with the surface more difficult, and with no obvious surface evidence of the oceans.

Although Titan possesses a large subsurface ocean, it also has an abundant supply of a wide range of organic species and surface liquids, which are readily accessible and could harbor more exotic forms of life. Further, Titan may have transient surface liquid water such as impact melt pools and fresh cryovolcanic flows in contact with both solid and liquid surface organics. These environments present unique and important locations for investigating prebiotic chemistry and, potentially, the first steps toward life.

Bodies such as Triton, Pluto, Ceres, and Dione are considered candidate ocean worlds based on hints from limited spacecraft observations. For other bodies, such as some Uranian moons, our knowledge is limited and the presence of an ocean is uncertain but they are deemed credible possibilities.

## 3. Ocean Worlds: Goals, Objectives, and Investigations

**FIG. 1.**
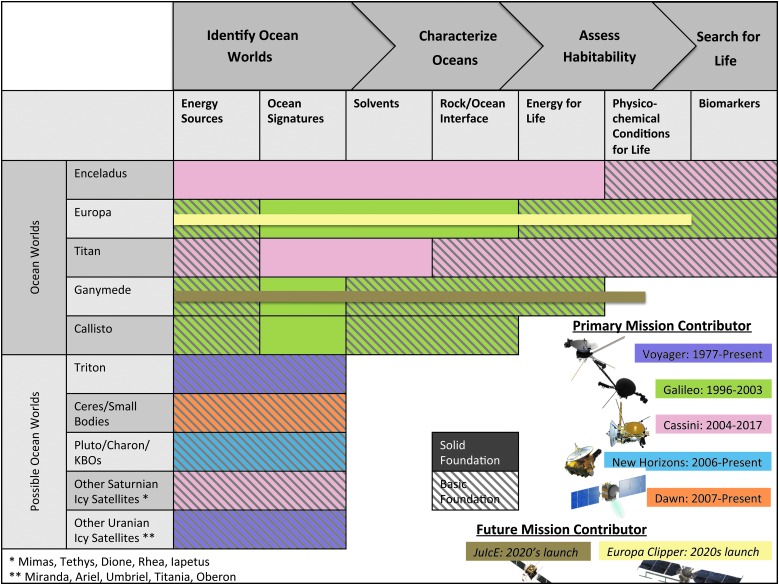
Investigations roadmap: demonstrating the state of knowledge for each (potential) target world. Colors represent the missions that provided the majority of information about each target. An evaluation on how well each target is understood for the various science objectives has been included: A solid color represents a solid foundation for addressing the science objective, whereas a hashed color represents only a basic foundation.

The overarching goal of an Ocean Worlds Program, as defined by the ROW team, is to: Identify ocean worlds, characterize their oceans, evaluate their habitability, search for life, and ultimately understand any life we find. This overarching goal naturally can be subdivided into four underlying goals, each of which has its own set of objectives and investigations (summarized in Table 1). Figure 1 demonstrates the state of knowledge of each objective, for the primary Solar System targets under discussion.

**Table 1.** Roadmaps to Ocean Worlds Goals, Objectives, Investigations

*Goal*	*Objective*	*Investigation*	*Spacecraft or R&A*
I. Identify ocean worlds in the Solar System	A. Is there a sufficient energy source to support a persistent ocean?	1. Is there remnant radiogenic heating?	R&A+spacecraft
		2. Is there gravitational energy from a parent planet or satellite?	
		3. Can the planet or satellite convert available tidal energy into heat?	
		4. Are the planet's or satellite's orbital or rotational properties favorable to tidal dissipation?	
	B. Are signatures of ongoing geological activity (or current liquids) detected?	1. Do signatures of geological activity indicate the possible presence of a subsurface ocean? (surface hotspots, plumes, crater-free areas, volcanoes, tectonics)	Spacecraft data required (Earth-based telescopic data could be adequate for some initial work)
		2. Are temporal changes observed at the body that would indicate the presence of a subsurface ocean?	
		3. Can the surface composition be linked with the presence of a subsurface ocean?	
		4. Is the signature of a surface liquid observed (*e.g.*, specular reflection)?	
		5. Does the body exhibit tidal and/or rotational evidence indicating the presence of a subsurface ocean?	
		6. Does the electromagnetic response of the body indicate the presence of a subsurface ocean?	
		7. Does the gravity and topography of the body indicate the presence of a subsurface ocean?	
		8. Is there an atmosphere or exosphere that could be linked with the presence of a subsurface ocean?	
	C. How do materials behave under conditions that are relevant to any particular target body?	1. What are the phase relations of materials composing ocean worlds at relevant pressures and temperatures?	R&A
		2. What is the composition and chemical behavior of materials composing ocean worlds?	
		3. What are the rheologic mechanisms by which material deforms under conditions that are relevant to ocean worlds?	
		4. How does energy attenuation/dissipation occur under conditions that are relevant to ocean worlds?	
		5. What are the thermophysical properties of materials under conditions that are relevant to ocean worlds?	
II. Characterize the ocean of each ocean world	A. Characterize the physical properties of the ocean and outer ice shell	1. What is the thickness, composition (including the presence of any organics), and porosity of the ice shell (crust) and how do these properties vary spatially and/or temporally?	R&A+spacecraft
		2. What is the thickness, salinity, density, and composition of the ocean? How do these properties vary spatially and/or temporally?	
		3. What are the drivers for, and pattern of, fluid motion within the ocean?	
	B. Characterize the ocean interfaces	1. Characterize the ice–ocean interface	
		2. Characterize the seafloor, including the high-pressure ocean–silicate interaction	
III. Characterize the habitability of each ocean world	A. What is the availability (type and magnitude/flux) of energy sources suitable for life? How does it vary throughout the ocean and time, and what processes control that distribution?	1. What environments possess redox disequilibria, in what forms, in what magnitude, how rapidly dissipated by abiotic reactions, and how rapidly replenished by local processes?	R&A
		2. (Where) is electromagnetic radiation available? In what wavelengths and intensity?	R&A+spacecraft
	B. What is the availability (chemical form and abundance) of the biogenic elements, how does it vary throughout the ocean and time, and what processes control that distribution?	1. What is the inventory of organic compounds, what are their sources and sinks, and what is their stability with respect to the local environment?	Spacecraft
		2. What is the abundance and chemical form of nitrogen, oxygen, phosphorus, sulfur, and inorganic carbon, what are their sources and sinks, and are there processes of irreversible loss or sequestration relative to the liquid environment?	Spacecraft
IV. Understand how life might exist in each ocean world, search for life, and understand the biology	A. What are the potential biomarkers in each habitable niche? (determine *what* we are looking for)	1. What can we learn about life in ocean worlds from studying the Earth?	R&A
		2. What niches for life are possible in ocean worlds?	
		3. What can we learn about life by understanding the history of ocean worlds from their formation to the present?	
		4. What should be our target indicators? (Life Detection Ladder)	
		5. How do we distinguish extant from extinct life in environments in which life might develop, and which timescales (*e.g.*, for metabolism, reproduction, dormancy) matter?	
	B *How* to search for and analyze data in different environments?	1. How can we look for life in an ocean world remotely (from orbit or during a flyby)?	R&A
		2. How can we look for life in an ocean world by using *in situ* (landed, underwater, plume) investigations?	
		3. How can we look for life in an ocean world with sample return science?	
		4. Which science operational strategies should be used to detect life in ocean worlds?	

R&A = Research and Analysis.

### 3.1. Goal I. Identify ocean worlds in the Solar System

Before sending spacecraft to a target body to search for life within the ocean, we must first demonstrate that an ocean exists. There are several questions that can be addressed to determine the presence of an ocean. For the confirmed ocean worlds (Europa, Enceladus, Titan, Ganymede, and Callisto), these questions have already been answered—or enough of the questions have been answered that the presence of an ocean is (reasonably) certain.

I.A. Is there a sufficient energy source to support a persistent ocean?

A.1. Is there remnant radiogenic heating?
A.2. Is there gravitational energy from a parent planet or satellite?
A.3. Can the planet or satellite convert available tidal energy into heat?
A.4. Are the planet's or satellite's orbital or rotational properties favorable to tidal dissipation?

Energy sources are perhaps the single most fundamental requirements, for the maintenance of a present-day ocean on an otherwise frozen world. The identification of ocean worlds, therefore, requires identification of possible energy sources. Both radiogenic heating (A.1) (*e.g.*, for Europa, Ganymede, Callisto, and Titan) and tidal energy (A.2) (*e.g.*, for Europa, Enceladus) play a role in sustaining oceans (Hussmann *et al.*, 2006). Available energy sources can be identified through either modeling or direct observation (or ideally a combination of the two). Theoretical modeling is an invaluable tool for predicting which bodies can sustain ocean worlds. Some models anticipated oceans on icy moons (*e.g.*, Europa) long before such oceans were ever actually detected (Lewis, 1971; Consolmagno and Lewis, 1978). However, modeling alone can lead to misleading results. In the case of Enceladus, theoretical models indicated that insufficient conversion of tidal energy to heat should occur within the Moon (Meyer and Wisdom, 2008; Roberts and Nimmo, 2008). Observations by the *Cassini* spacecraft have demonstrated that such heating does, in fact, occur, but the Moon emits an order of magnitude more energy (at the present time) than theoretical models predicted (Spencer *et al.*, 2006; Howett *et al.*, 2011). Identifying the sources of Enceladus' energy and its transient versus long-lived nature is an open and active area of research.

Evaluating sources of energy requires addressing, at a minimum, the four sub-questions listed earlier. For the largest satellites, remnant radiogenic heating may be sufficient to maintain an internal ocean (A.1), depending on the initial radiogenic content of the rock component, and the state of the overlying ice shell (Hussmann *et al.*, 2006; Schubert *et al.*, 2010). For smaller bodies (*e.g.*, Enceladus), dissipation of tidal energy is critical (Schubert *et al.*, 2010). Dissipation of tidal energy requires the presence of a parent planet or satellite with sufficient gravitational energy to deform the body (A.2). Pluto and Charon lack a source of such energy, so the energetics that permit a long-lived ocean within these bodies are still in question (Nimmo and Spencer, 2015).

In addition, a body's orbit and/or rotation must be favorable to tidal dissipation, possibly through a high eccentricity (*e.g.*, Europa) (Sotin *et al.*, 2009), libration (Wisdom, 2004), or obliquity (*e.g.*, Triton) (Nimmo and Spencer, 2015) (A.4). Pluto and Charon lack a source of such energy because their orbits have evolved toward circularization (*i.e.*, zero eccentricity). However, these two requirements are insufficient to ensure internal oceans, as the planet or satellite must be able to convert available tidal energy to heat (A.3) (Tobie *et al.*, 2005). This is demonstrated by the satellite Mimas, which, despite its high eccentricity, dissipates little tidal energy, likely because its interior has remained cold since shortly after its formation (McKinnon, 2010). The complex feedback between the orbital/rotational evolution of potential ocean worlds (A.4) and their internal structure (A.3) requires careful theoretical modeling (Ojakangas and Stevenson, 1989; Showman *et al.*, 1997; Hussmann and Spohn, 2004).

I.B. Are signatures of ongoing geological activity (or current liquids) detected?

B.1. Do signatures of geological activity indicate energy sources to maintain a subsurface ocean? (surface hotspots, plumes, crater-free areas, volcanoes, tectonics)
B.2. Are temporal changes observed at the body that would indicate the presence of a subsurface ocean?
B.3. Can the surface composition be interpreted as consistent with the presence of a subsurface ocean?
B.4. Is the signature of a surface liquid observed (*e.g.*, specular reflection)?
B.5. Does the body exhibit tidal and/or rotational evidence indicating the presence of a subsurface ocean?
B.6. Does the electromagnetic response of the body indicate the presence of a subsurface ocean?
B.7. Does the gravity and topography of the body indicate the presence of a subsurface ocean?
B.8. Is there an atmosphere or exosphere that could be linked with the presence of a subsurface ocean?

Over the past three decades, numerous techniques have been developed for assessing whether subsurface oceans are present on icy worlds. In some cases, investigation of a satellite's surface is sufficient to infer the possible presence of an ocean below (Pappalardo *et al.*, 1999). Recent or ongoing geological activity, such as a young surface shaped by tectonics, hotspots, and plumes, is indicative of a warm interior that can potentially sustain an ocean (B.1). For example, the plume of Enceladus, along with the young, crater-free terrain and warm fractures from which it emanates are strong indicators of an ocean, even in the absence of other geophysical data (Porco *et al.*, 2006). Likewise, surface change (B.2) could indicate ongoing geological activity, again requiring a warm interior.

Surface composition (B.3) can also suggest a subsurface ocean through the presence of chemical species originating in an ocean (Hand and Carlson, 2015). In the case of Europa, consensus on the origin of surface chemical species remains uncertain (since irradiation can modify the molecular composition), for example, from chlorides to sulfates (Carlson *et al.*, 2009); however, improved spectral and spatial resolution data are likely to resolve the question. In the case of Ceres, the recent emplacement of salt-rich subsurface material in several places on the surface (*e.g.*, Ahuna mons, Occator crater) is interpreted as evidence for briny liquids at depth (DeSanctis et al., 2016; Ruesch *et al.*, 2016). In rare cases where liquids may be present on a body's surface rather than, or in addition to, within its interior (*e.g.*, Titan), liquids can be inferred through their optical and radar properties (Stofan *et al.*, 2007; Brown *et al.*, 2008; Turtle *et al.*, 2009) or by specular reflection (B.4) (Wye *et al.*, 2009; Stephan *et al.*, 2010).

Not all ocean worlds reveal their present oceans in their surface characteristics. Both Ganymede and Callisto have internal oceans, but to our knowledge, their surfaces are currently inactive (Moore *et al.*, 2004; Pappalardo *et al.*, 2004). For these worlds, and to confirm oceans on geologically active worlds, a number of geophysical measurements can be used to identify present-day oceans. In many cases, oceans can be revealed by the orbital and rotational state of a body (B.5), if it can be measured carefully enough. For example, the magnitude of Enceladus' physical libration requires the presence of a global ocean (Thomas *et al.*, 2016). Titan's subsurface ocean is also revealed by differential rotation of its outer shell, which must be decoupled from its interior (Lorenz *et al.*, 2008). For systems with a strong, inclined magnetic field (*e.g.*, the Jupiter system), the electromagnetic response of the body, after correction for ionospheric background, provides a strong indication of an internal ocean (B.6), as demonstrated for Europa, Ganymede, and Callisto (Kivelson *et al.*, 1999, 2000, 2002). With sufficient flybys, gravity data can also indicate the presence of an ocean (Iess *et al.*, 2014; McKinnon, 2015), especially when coupled with detailed topography (B.7), as recently demonstrated for Dione (Hemingway *et al.*, 2016).

Implicit in the earlier discussion is the necessity of spacecraft data to characterize the surfaces of bodies and acquire geophysical data. In some cases, ground-based data can aid our understanding. This is especially true for the monitoring of a satellite's exosphere for potential plume activities (as in the case of Europa, Roth *et al.*, 2014; Sparks *et al.*, 2017) (B.8). However, unambiguously identifying ocean worlds requires detailed *in situ* investigations.

I.C. How do materials behave under conditions that are relevant to any particular target body?

C.1. What are the phase relations of materials composing ocean worlds at relevant pressures and temperatures?
C.2. What is the composition and chemical behavior of materials composing ocean worlds?
C.3. What are the rheological mechanisms by which material deforms under conditions that are relevant to ocean worlds?
C.4. How does energy attenuation/dissipation occur under conditions that are relevant to ocean worlds?
C.5. What are the thermophysical properties of material under conditions that are relevant to ocean worlds?

This is a “basic research” area and is discussed further in this article (see Sec. 6).

### 3.2. Goal II. Characterize the ocean of each ocean world

Once an ocean has been demonstrated to exist in any ocean world, an immediate priority will be to understand the physics and chemistry of that ocean to gain knowledge of how the system functions and how that, in turn, informs our assessment of its potential habitability. Such characterization permits identification of appropriate future life detection experiments, if warranted. To characterize the ocean, the following objectives and investigations must be addressed.

II.A. Characterize the physical properties of the ocean and outer ice shell

A.1. What is the thickness, composition, porosity, and rheology of the ice shell (crust) and how do these properties vary spatially and/or temporally?
A.2. What is the thickness, salinity, density, and composition of the ocean? How do these properties vary spatially and/or temporally?
A.3. What are the drivers for, and pattern of, fluid motion within the ocean?

Properties of the external ice shell (A.1) and its outer surface environment will not only influence ocean world habitability but also determine the extent to which biosignatures may be expressed on the surface and impact the design of any future exploration (Chyba and Phillips, 2001; Figueredo *et al.*, 2003; Hand *et al.*, 2009). Ice on the Earth is a rich habitat; should life arise in ocean worlds, then their ice shells may be similarly rich in habitable environments, given proper conditions. Of specific interest are those properties that affect transport processes through the ice shell, both thermal and physical, as they play a crucial role in the evolution and dynamics of the world. Material transport between the surface and subsurface is potentially crucial for mediating energy and nutrient flow, as well as ocean pH, oxidants, and other factors that govern habitability of the shell itself (potentially rife with brine zones, water pockets, and habitable ice grain boundaries) and that of the ocean below. The overall thickness of the ice shell, and any spatial variability, will determine the propensity and timescales for ocean–surface interaction and what modes of transport are possible (McKinnon, 1999; Gaidos and Nimmo, 2000; Pappalardo and Barr, 2004; Katterhorn and Prockter, 2014).

**FIG. 2.**
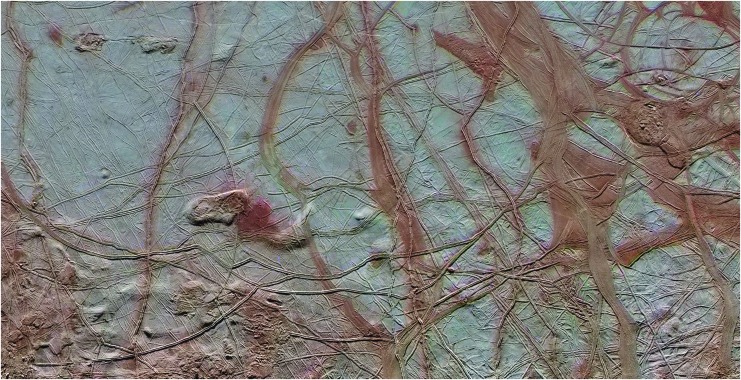
Enhanced-color image of a region on Europa's surface, highlighting an intricate network of spots, ridges, and lenticulae that appear to demonstrate communication with an underlying subsurface ocean. Image credit: NASA/JPL.

In the cold, brittle shells (or stagnant lids of thicker shells) fracture processes will likely dominate as the viscosity of ice at low temperatures greatly hinders ductile deformation, and for any geothermal and tidal heat production regime there is a corresponding limit to when the shell will transition from transferring heat through conduction to doing so by convection. Fractures in brittle conductive shells may provide a direct path for material exchange from the subsurface to the surface; however, downward material motion is poorly understood and perhaps unlikely, as in the case of Enceladus' south polar terrain (Glein *et al.*, 2015). For thicker, more temperate ice shells, large-scale convective motion of the ice and entrained or endogenic liquids can provide a geologically rapid transport mechanism between the underlying ocean and the upper portions of the shell (McKinnon, 1999; Pappalardo and Barr, 2004; Schmidt *et al.*, 2011; Peddinti and McNamara, 2015). For an archetypical layered shell, like that of Europa (Fig. 2) with a brittle stagnant lid overlying a convecting ice mantle, a combination of tectonic processes (such as subsumption/subduction) (Katterhorn and Prockter, 2014) and convective overturn could produce a continuous chemical cycling of ocean-derived reductants to the surface and oxidized materials from the surface back into the underlying ocean.

Life as we know it depends on the availability of redox reactions, which makes understanding planetary scale and local transport cycles crucial in quantifying the habitability of ocean worlds. In quantifying large-scale transport rates, knowledge of the ice shell composition and basal entrainment rates are needed. The chemical cycling discussed earlier relies on the presence of impurities within the ice shell. The level and location of these impurities dictates the flux of oxidants and reductants to and from the ocean, respectively, and can also aid or hinder convective overturn, as well as govern intra-shell habitability (Zolotov and Kargel, 2009).

Oceans exist as solutions of water and dissolved compounds. The fractional crystallization of this material on freezing into pure ice and rejected brine leads to a variety of physicochemical effects, including the accretion of ocean-formed ice (Vance and Goodman, 2009; Soderlund *et al.*, 2014; Willis *et al.*, 2016). On the Earth, this is manifested as porous “slushy” layers near the solid–liquid phase interface. This is exemplified by the basal surface of sea ice, which forms brine pockets and channels leading to a residual salinity profile throughout the ice, as well as by “marine ice” that forms beneath thick permanent ice such as ice shelves. Understanding the physicochemical processes that govern physical and chemical evolution within the ice shell and at its base can give insight into the compositional stratigraphy of ice shells, the reductant entrainment rate, and the temporal evolution of the shell, as well as offer insights into whether biological signatures from the ocean are likely to be preserved within bulk ice. Because these impurities modify the properties of the ice, they can also impact the phase distribution within the shell. If concentrated deposits of impurities exist within the ice shell, the local eutectic point can be drastically reduced. If these deposits are heated above their eutectic point, melting occurs accompanied by a change in volume. This mechanism has been proposed as a method for producing chaos terrain on Europa (Schmidt *et al.*, 2011) and could aid in chemical transport on ocean worlds.

Basic characteristics of oceans (*e.g.*, salinity, density) are critical for ultimately understanding habitability. Pressure increases with depth and will vary as a function of both the gravitational acceleration of the ocean world in question and the physical properties (*e.g.*, compressibility) of the dominant liquid phase (A.2). At the top of the ocean, in any icy ocean world, we can imagine a near-isothermal surface at the ice–water interface (Melosh *et al.*, 2004). Densities of water masses will then vary primarily as a function of salinity. In an ocean world that has active ocean–atmosphere interactions, as on the Earth, significant variations in salinity can be established through evaporation–precipitation cycles. On ice-covered ocean world systems, by contrast, introduction of any variations in salinity may arise from brine exclusion during freezing at the outer ice–water interface or, like any thermal inputs, through water–rock interactions at the seafloor—the same processes that are of interest in terms of habitability and the astrobiological search for life because of their potential to host and sustain chemosynthetic ecosystems.

On the Earth, the salinity of the oceans (*i.e.*, the total concentration of dissolved salts) is relatively uniform at about 3.5% by mass, reflecting the relatively rapid overturning time for the entire ocean volume. Further, the proportions of dissolved salts within the Earth's oceans (Na^+^, Cl^−^ > Mg^2+^, SO_4_^2−^ > Ca^2+^, K^+^, HCO_3_^−^, Br^−^) are in almost constant proportions everywhere. Importantly, however, the abundances of these species in the Earth's oceans are decoupled from their abundances on the Earth as a whole, reflecting fractionations that can be used to infer the key “weathering” processes on our planet—that is, the chemical reactions taking place between the oceans and major rock types. Because the same telltale phenomenology could be occurring in extraterrestrial oceans, it will also be important to not only characterize the total salinity of the oceans and how uniform the salinity field might be (A.2) but also determine what the relative proportions of different chemical species are within that ocean.

Changes in salinity are likely to play at least as important a role in driving physical circulation on other ocean worlds as does sunlight in driving circulation in the Earth's oceans. But understanding the chemical composition of that ocean will also represent an extremely effective way of understanding what the dominant lithologies (ultramafic/chondritic, basaltic, silicic) are at the underlying seafloor. This, in turn, will provide immediate insights into the geological evolution of that ocean world and what water–rock interactions are dominant, which are critical to understanding the planet's habitability. Changes in salinity and/or composition across the ocean world may also help reveal where any major seafloor inputs arise.

On the Earth, our oceans are in constant motion but the primary drivers—solar insolation and ocean circulation (A.3)—may be absent on some icy worlds. For example, sunlight falling on the surface of our planet drives ocean circulation through atmospheric circulation. Frictional forces transfer energy from winds at the base of the atmosphere, driving circulation of the uppermost ocean. Heating of the ocean surface from sunlight also helps to drive the deep thermohaline circulation on the Earth. Heating near the equator drives processes of evaporation and precipitation across the air–sea interface; meanwhile, cooling effects at the poles lead to freezing of relatively fresh water-ice at the surface, leaving behind relatively salty, hence dense, ocean waters that sink, leading to deep ocean ventilation—that is, mixing of the entire deep ocean system on timescales that are extremely short (order 1000–2000 years) when compared with the age of the planet. In other ocean worlds with large and suitably deep liquid bodies that connect to an atmosphere (*e.g.*, Titan), similar processes could occur.

By contrast, in entirely ice-covered ocean worlds (*e.g.*, Europa, Enceladus) where solar heating is absent, top-driven thermal circulation is unlikely. Rather, the ice–water interface at the top of the ocean is likely to be isothermal, assuming the ice-cap is too ductile to sustain any significant sub-ice topography that could lead to changes in pressure sufficient to drive downwelling of water connected to the ice. Similarly, only if there are significant variations in the topology of the underside of an ice shell is the case likely to arise in which localized variability in brine exclusion during freezing (the accumulation of relatively dense salty water when pure water-ice is frozen out) is any downward convection likely to be driven by salinity (J. Marshall, MIT, pers. comm.). Otherwise, any thermally driven circulation will more probably be driven from below (Goodman and Lenferink, 2012).

A second process that impacts ocean circulation on the Earth is planetary rotation. On any other body with a global-scale ocean, rotation will be expected to dominate circulation processes. In these thin rotating shells, the competition between rotational and inertial forces likely results in Hadley-like circulation, where most thermal energy rises at the equator and sinks at the mid-latitudes, mixing the ocean and moving water in thick meridional jets (Soderlund *et al.*, 2014). These convective and transport mechanisms can influence ice accretion and melting at the ice–ocean interface, as well as govern the transport of nutrients and/or biosignatures through the ocean system.

Tidal modulation of the shell may also contribute to the ocean convection and transport dynamics (Tyler, 2008). As on the Earth, tidal processes may also lead to important mixing within an ocean—even in an icy ocean world. This will be highly dependent on the gravitational interaction of any ocean world and its adjacent moons. The same processes seen on the Earth are also important for Europa, for example, and may be for Enceladus and Titan, too. On the Earth, within currents flowing north or south, toward or away from the equator, the fluid flow is decoupled from the rigid underlying seafloor. As a parcel of water travels to higher latitudes, the rotational velocity of the underlying (eastward moving) seabed decreases and, hence, there is a relative motion of the ocean compared with the seafloor that is progressively toward the east at higher latitude (or to the west for currents flowing toward the equator). The exception arises when deep ocean currents intercept the seafloor mid-ocean ridges and both topographic steering and significant vertical mixing result (Toole and Warren, 1993; Scott *et al.*, 2001). Similar mixing behavior within other ocean worlds could also affect such global calculations noted earlier, but the importance of these processes will scale with the depth of the ocean, the topography of the seafloor, the drivers for poleward currents, and the rotational velocity of the ocean world under consideration.

II.B. Characterize the ocean interfaces

B.1. Characterize the ice–ocean interface
B.2. Characterize the seafloor, including the high-pressure ocean–silicate interaction

Interfaces are a critical component of habitability because they can present sharp gradients in the materials (*e.g.*, CHNOPS) and energy sources (*e.g.*, oxidants and reductants) required for life. For ocean worlds, two significant interfaces are recognized: water–ice (B.1) and water–rock (B.2). However, these interfaces are not limited to the obvious boundaries between solid and liquid media. Sediment porewater, subsurface aquifers, ice brine channels, and melt lenses are all examples of interfaces existing away from the major zone of transition. These features are particularly significant because they extend the surface area of the water–rock or ice–water interface far beyond what is suggested by obvious, large-scale boundaries. For some bodies, ice brine channels and melt lenses also constitute interfaces that are much closer to the surface (and thus potentially easier to study) than would otherwise be the case.

On the Earth, life is strongly associated with interfaces, prominent examples being the microbial mats that grow atop marine and aquatic sediments on the underside of sea ice and at and within young ocean crust. Association with these interfaces allows the cells comprising these communities to maintain an optimal position with respect to gradients of nutrients and energy in a turbulent environment. Because all life on the Earth derives energy either from sunlight or from the reaction energy of chemical redox pairs (either present in the environment or produced by cells from energy in the environment), identification of a redox gradient is highly relevant to any search for life in ocean worlds. The fundamental nature of planetary material results in a partitioning of oxidants and reductants at the planetary surface and interior, respectively. As on the Earth, interfaces that constitute boundaries between the surface and interior may be ideal habitats. On an ocean world, liquid water can extend these boundaries by transporting oxidants and reductants, placing them in close enough proximity to fuel life-sustaining processes. Solid surfaces can also provide a location for the concentration of nutrients, an attachment point for cells, and a stable habitat for life (potentially) in a habitable subsurface ocean.

The ice–ocean interface (B.1) can be studied indirectly through measuring a combination of surface topography (whose physical support depends on the thickness and mechanical properties of the ice below), the tidal flexure of the ice shell (that depends on its thickness), and magnetic signature (which places constraints on the total ice thickness and the conductivity of the ocean once the shell thickness is known). The ice–ocean interface can be directly observable by radar sounding for sufficiently thin shells. For Europa, this characterization could be the direct observation of the interface by the reflection of radar waves by the ice–ocean interface (radar waves are reflected by water), or through the damping of or loss of signal from warm, briny, or salty ice in an otherwise continuous ice shell.

To date, theoretical modeling with observational constraints (*e.g.*, heat flow measurements, spectroscopy) supplies basic information about interior processes that would occur at interfaces. The chemical species observed on planetary surfaces can indirectly inform us about interfaces occurring below the surface. Fine-scale interfaces, such as the brine pores or crystal boundaries in ice, cannot be observed remotely but their structure and nature can be predicted from laboratory and Earth analog studies. Marine ice, accreted saline ice that forms under moderate pressure at the base of ice shelves, is one example of an understudied ice type that may mimic some ices in ocean worlds. Relevant interfacial features of ice microstructure, including porosity and connectivity, have been insufficiently explored in laboratory studies under the temperature, pressure, and salinity conditions that are relevant to ocean worlds. Because interfaces are important to life, many interface-dependent organisms on the Earth have evolved the capacity to modify interfaces. This capability may invalidate laboratory and modeling efforts that seek to characterize interfaces under strictly abiotic conditions. On the Earth, these interfaces can be explored by oceanographic moorings, AUV/ROV/HOV exploration, and other *in situ* techniques as well as through the study of direct samples. In the foreseeable future, such exploration may become feasible in ocean worlds.

When considering the habitability of an ocean world, especially one far from the Sun where photosynthesis is unlikely to be effective, a key issue is the nature of the underlying seafloor and water–rock interactions (B.2). On the Earth, where plate tectonics is the norm, fluid–rock interactions occur under a range of incompletely studied conditions of pressure and temperature. For example, where it was initially assumed that the Earth's entire ocean floor was made up of basalts, we now know that lithologies including basaltic andesites and even more silicic calc-alkaline lavas can occur in back-arc and arc settings at convergent margins. Conversely, ultramafic as well as basaltic rocks can be exposed to water–rock interactions in subduction-zone trenches, transform faults, fracture zones, and at slow and ultra-slow spreading mid-ocean ridges. Although the full diversity of settings observed on the Earth may not be repeated on any single other ocean world, an advantage provided by our home planet is the opportunity to conduct a wealth of analog studies spanning a broad range of conditions that may be relevant to other ocean worlds in terms of seafloor pressure, maximum water temperatures at those pressures, and interactions with different rock types (reactions that can both result in and be diagnosed from ocean chemical compositions).

**FIG. 3.**
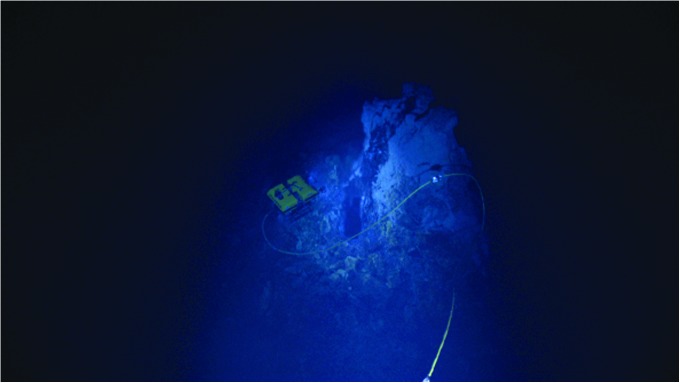
ROV Little Hercules inspecting the 8-m-tall Von Damm hydrothermal spire, Mid Cayman Rise. The Von Damm site is an ultramafic-influenced seafloor system that serves as one plausible candidate for conditions inferred at the seafloor of Enceladus (McDermott *et al.*, 2015; Waite *et al.*, 2017). Photo courtesy of NOAA. NOAA, National Oceanic and Atmospheric Administration.

**FIG. 4.**
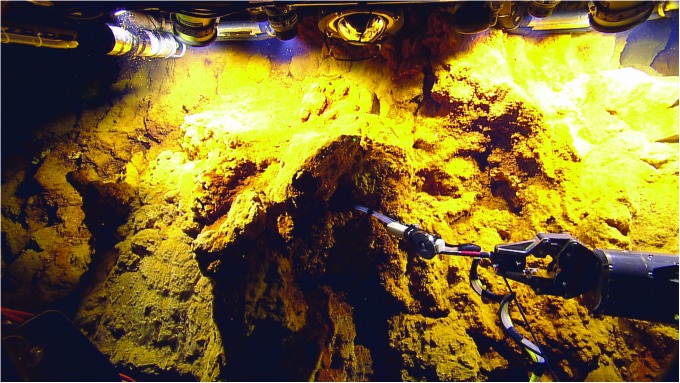
ROV Hercules sampling for hydrothermal fluid compositions and associated microbiology at the Dragon Cave vent, Loihi seamount, during the SUBSEA program, a model NOAA/NASA co-funded R&A collaboration with dual focus on Ocean Exploration and Ocean World analog studies. Photograph courtesy of Ocean Exploration Trust. R&A, Research and Analysis.

From an astrobiological perspective, the nature of geological processes that may be active at the seafloor and, specifically, what processes might drive the underlying heat flow are critical. For example, recent studies by Hsu *et al.* (2015) and Sekine *et al.* (2015) have suggested that hydrothermal venting on Enceladus may be relatively low temperature (50–200°C), and Glein *et al.* (2015) suggested that the ocean composition may be rather Na-rich like the Earth, but significantly more alkaline. Such evidence suggests several candidate Earth analogues: ultramafic intermediate temperature hydrothermal systems such as the Lost City or Von Damm hydrothermal fields (Kelley *et al.*, 2001; McDermott *et al.*, 2015) or the Loihi Seamount off Hawai‘i (German *et al.*, 2018) (Figs. 3 and 4).

In contrast, tidal modeling for Europa (Sotin *et al.*, 2009) has provided evidence that dissipation of energy may occur predominantly in the liquid water ocean, leading to flexing in the outer ice shell but relatively little impact on the silicate interior (quite distinct from the abundant volcanism seen on its sister moon Io). If so, then the dominant thermal processes at the seafloor of Europa may be related less to active magmatism and volcanism and more to passive cooling (*e.g.*, conductive) of radiogenic heating of the solid interior. In the latter case, progressive cooling might still be expected to lead to thermal contraction at the seafloor and cracking to allow fluid circulation to penetrate beneath the seafloor (Vance *et al.*, 2007); such circulation might be closer to forms of circulation that have only been reported in fossilized form from the Earth's deep fracture zones, to date (Bonatti *et al.*, 1978) but the resulting generation of reduced hydrogen from serpentinization may equal or exceed potential fluxes from high-temperature hydrothermal activity (Vance *et al.*, 2016).

The style and vigor of any hydrothermal circulation might vary significantly depending on the nature of the processes happening at and beneath the seafloor of any ocean world. In an isothermal ocean, even a weakly buoyant hydrothermal system might still penetrate all the way to the ocean surface but the interesting possibility arises that the most pertinent analog settings on the Earth to inform our study of other ocean worlds might be in tectonic settings such as fracture zones or subduction zones that have been completely or, at the very least, largely overlooked on our own planet. Indeed, even the Earth's mid-ocean ridges (which have received an increased level of attention since the time of the Viking Landers) remain 80% unexplored (Beaulieu *et al.*, 2015). Isolated volcanoes that are more reminiscent of what is seen elsewhere in our Solar System (Mars, Io) are also not only known to occur abundantly across the Earth's ocean floor but also remain largely unexplored for seafloor fluid flow and habitability (German *et al.*, 2018).

Key parameters to consider for the seafloors of other ocean worlds, therefore, would include a search for anomalies in both topography (the depth to the ocean floor) and heat flow (evidence for convection vs. thermal conduction), which could provide important evidence for geological processes operating on that ocean world (organized planetary-scale plate tectonics, isolated volcanism, and seafloor fracturing). Assuming slow rates of erosion and/or thermal relaxation of the planet's solid interior, the shape of the seafloor alone would provide important evidence for past geological activity, even if those processes are no longer active in the modern day. By adding heat flow or seismic investigations to such a study, however, one would also gain insights, immediately, into whether such processes might be ongoing.

### 3.3. Goal III. Characterize the habitability of each ocean world

III.A. What is the availability (type and magnitude/flux) of energy sources that are suitable for life? How does it vary throughout the ocean and time, and what processes control that distribution?

A.1. What environments possess redox disequilibria, in what forms, in what magnitude, how rapidly dissipated by abiotic reactions, and how rapidly replenished by local processes?
A.2. (Where) is electromagnetic or other energetic radiation available? In what wavelengths or energy and intensity?

Life on the Earth utilizes, as sources of energy, light in the visible to near-infrared (IR) wavelength range and the chemical energy released in specific (mostly oxidation-reduction) chemical reactions; surface irradiation may alternatively be an effective source of oxidants at icy moons in giant planet magnetospheres (Cooper *et al.*, 2001, 2009). The present understanding of biological energy metabolism indicates that chemical energy sources must satisfy discrete minimum requirements for both Gibbs energy change (ΔG) and power (flux of energy through time) to be useful. Light energy also must satisfy a discrete minimum requirement for flux (corresponding to light intensity; the requirement equivalent to ΔG is easily satisfied in any of the parts of the wavelength range used by life). In addition, the flux of energy constrains, in a direct relationship, both the maximum rate of new biomass synthesis (productivity) and the maximum quantity of standing biomass that can be sustained in steady state. That is, environments having greater energy flux can potentially support more abundant life and might, therefore, be better targets for life detection. Importantly, at Europa, high oceanic fluxes of seafloor reductants from low- or high-temperature hydrothermal activity (Vance *et al.*, 2016) may be complemented by oxidants generated by surface radiolysis (Hand *et al.*, 2007), though it is as yet unclear as to how much oxygen reaches the ocean. The total redox flux to Europa's global ocean may exceed fluxes in other ocean worlds that have less active ice and less surface radiation, although too much abiotic oxygen could be detrimental to life, confirming calls for understanding the ice's geology, oxidation state, and corresponding rates of delivery of surface materials into the ocean.

**FIG. 5.**
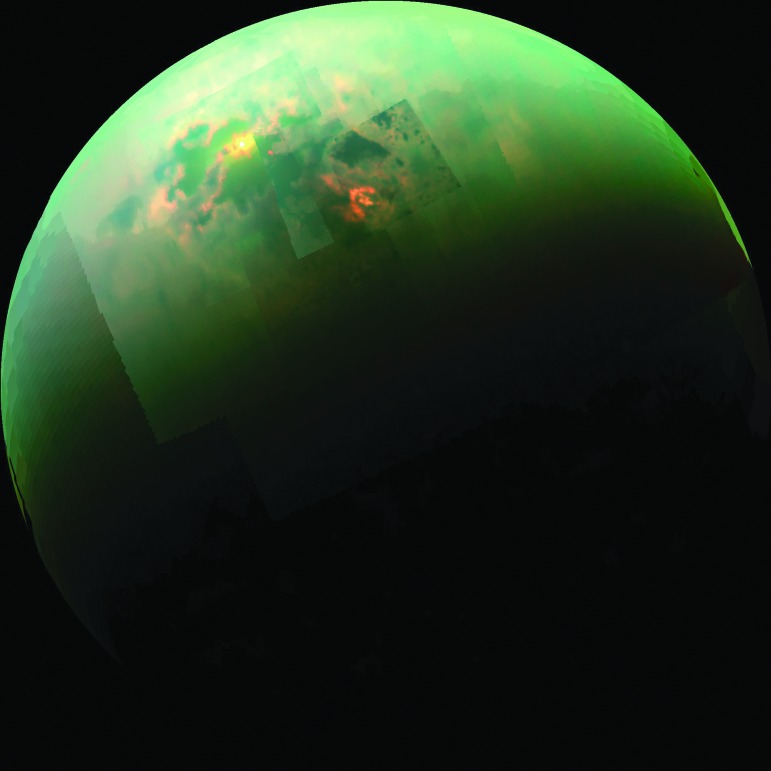
This near-infrared, color mosaic from NASA's Cassini spacecraft shows the sun glinting off of Titan's north polar seas. Credit: NASA/JPL-Caltech/University of Arizona/University of Idaho.

Investigations A.1 and A.2 call for characterization of the availability of the two forms of energy known to be utilized by life on Earth (A.1: chemical; A.2: light). For A.1, spacecraft observations that constrain the concentrations of redox-active species within the liquid environment will support calculation of Gibbs energy yields (ΔG) associated with specific redox couples, thereby identifying metabolisms that satisfy the biological ΔG requirement. Assessment of energy flux will require spacecraft observations that constrain the rate of delivery of specific chemical species into the liquid environment—for example, delivery of oxidants into an ocean by overturn of surface ice and corresponding delivery of reductants by water–rock reaction. For A.2, observations or models are required that constrain the spectral character and intensity of light available within the liquid environment. In general, a kilometer-thick ice cover will preclude solar influx, but a transiently or locally thinner ice cover and black body radiation (*e.g.*, from hydrothermal vents) might allow for some introduction of light into liquid water habitats. Moreover, Titan's hydrocarbon lakes (Fig. 5), should they prove to represent a solvent that is suitable for life, receive direct solar irradiation.

III.B. What is the availability (chemical form and abundance) of the biogenic elements? How does it vary throughout the ocean and time, and what processes control that distribution?

B.1. What is the inventory of organic compounds? What are their sources and sinks, and what is their stability with respect to the local environment?
B.2. What is the abundance and chemical form of nitrogen, oxygen, phosphorus, sulfur, and inorganic carbon? What are their sources and sinks, and are there processes of irreversible loss or sequestration relative to the liquid environment?

The biochemistry of life on the Earth is built around a core of elements—C, H, N, O, P, and S (CHNOPS)—that are required by all known organisms, as well as a variety of other elements (*e.g.*, specific transition metals) that are required by specific subsets of life. Biominerals, including silicon or calcium, are also important to life. In earthly environments where life's requirements for water and energy are abundantly met—for example, the sunlit portions of the Earth's aquatic environments—the distribution of these elements (notably N and P, together with the micronutrient Fe) can directly limit the abundance and productivity of life. In harsh surface irradiation environments, as on Europa's trailing hemisphere, where organic molecules are quickly destroyed when fully exposed, the residual elemental composition could still provide biosignatures. Each of these elements can be incorporated into a diversity of chemical forms, some of which may be less accessible or inaccessible to biology.

Investigations B.1 and B.2 seek to establish the availability of these elements, in terms of both chemical form and abundance, and to characterize the processes that govern that availability. B.1 emphasizes the importance of organic compounds, whose presence could be indicative of the stability of specific chemical bonds or functional groups in the host environment, or which might plausibly serve as feedstock for biochemistry. Spacecraft observations that constrain the abundance, molecular character, and distribution in chemical space of organics within the liquid environment will directly support this investigation. B.2 targets the availability of the remaining biogenic elements and of inorganic carbon. Spacecraft observations that constrain the abundance and chemical form of compounds bearing these elements in the liquid environment will directly support this investigation.

### 3.4. Goal IV. Understand how life might exist at each ocean world, search for life, and understand the biology

IV.A. What are the potential biomarkers in each habitable niche? (determine *what* we are looking for)

A.1. What can we learn about life in ocean worlds from studying life on the Earth?
A.2. What niches for life are possible in ocean worlds?
A.3. What can we learn about life by understanding the history of life in ocean worlds?
A.4. What should be our target indicators?
A.5. How do we distinguish extant from extinct life in environments in which life might develop, and which timescales (*e.g.*, for metabolism, reproduction, dormancy) matter?

The Earth is the only planet yet where life is known to exist. Analog studies of life in the Earth's oceans and other habitable niches provide our only anchor for extrapolations to other ocean-bearing worlds (A.1). Examples of what can be learned from such studies include: (1) the range of physical (*e.g.*, temperature, pressure, radiation levels) and chemical (*e.g.*, pH, redox, salinity/water activity, major/trace elemental abundances) conditions that life tolerates; (2) whether known extremophiles exist at the temperatures, pressures, pH, radiation levels, etc. found on other ocean worlds; (3) whether there exist areas on the Earth that do not support life (and why); (4) how long it takes ecosystems to colonize a given environment, or adapt/evolve to changing physicochemical conditions; (5) the metabolic diversity that might be expected in ocean world environments; and (6) the amount of potential biomass that could be sustained in a given ocean world.

Niches for extant life (A.2) must provide a solvent, energy, and nutrients for a sufficient amount of time. By definition, ocean worlds provide a solvent, and the materials that planets form from can be expected to contain the necessary nutrients. Lacking sunlight, bioavailable energy requires the co-location, in chemical disequilibrium, of electron donors and acceptors. Determining which metabolic strategies are possible, which dominate, and what their spatiotemporal distribution is involves finding out which electron donors and acceptors are present, what their abundances are, whether there exist mechanisms to bring them together, and the quantification of sources, sinks, and their variation in space and time.

Our current knowledge of biology is entirely based on life on the Earth, but finding life elsewhere would provide other data points to infer universal properties of life. Key considerations include the emergence of life, how long a world has been inhabited, whether (and which) evolutionary pressures might have driven or prevented life's diversification, and how geological and environmental changes could affect habitability over time (A.3).

Ideal target indicators (A.4) are specific to life (not found in abiotic systems), universal (not limited to life on the Earth), and easy to detect. To date, we do not know of any single indicator that satisfies all three criteria. Darwinian evolution and reproduction may be the only specific and universal indicators, but we cannot measure them even for most of life on the Earth, which we cannot culture. Growth (concurrent life stages) and activity (motility, feeding, biofilm formation) are specific, but they may not be universal. Evidence for metabolism (isotopic fractionations from abiotic values, co-location of electron donors and acceptors) can be more easily detected, but its lack of specificity requires excellent contextual knowledge. Functional molecules (nucleic or amino acid polymers, pigments) are highly specific and easy to detect, but they may not be universal. Potential biomolecule components (nucleic or amino acids, lipids, sugars) with structural preferences (nonrandom chirality, carbon number, or trace element compositions) may be universal and easy to detect, but they have low specificity.

Any extant life in ocean worlds (A.5) is likely to be present as single-celled prokaryotes (and potentially eukaryotes). (This is also discussed in IV.B.2 below.) Micrometer-scale imaging can confirm the presence of cells irrespective of their chemistry, and fluorescence imaging with dyes that are specific to biochemicals (*e.g.*, lipids, proteins, nucleic acids, sugars) can assess the co-location of chemicals that are relevant to life and cell-like features. The required resolution for such imaging should be the same in ocean worlds with liquid water as on the Earth: The upper size limit for single-celled organisms is set by diffusion rates that are similar in liquid water everywhere, and the lower limit is set by the need to contain a self-replicating genome and the chemistry required to manufacture cellular proteins.

As on the Viking missions, metabolism can be observed by supplying electron donors/acceptors and labeled nutrients to a sample, and monitoring abundances of labeled products over time. This approach would benefit from simultaneous micro- and macroscopic imaging of the reaction volume. Microscopic imaging could detect growth, reproduction, motility (easily distinguished from Brownian motion), and taxis, and assess their correlation with chemical measurements of metabolic activity. Macroscopic imaging could perform the equivalent of colony counting. Rapid changes in activity can be stimulated by modest temperature changes or nutrient addition, although obvious changes, such as the regrowth of flagella from dormant bacteria, can take several hours.

On the Earth, microevolution can be very rapid in single-celled organisms. Organisms with random mutations might develop into distinct populations with different nutrient preferences. Thus, evolution might be detected within days or weeks, by supplying a sample container heterogeneously with a variety of chemical stimuli and measuring cell concentrations in different regions. Short of being able to induce evolution, a proxy is the observation of multiple species in a population, likely to arise in the presence of different habitable niches. Microbial diversity might be detected with short-term observations of differences in activity levels with different nutrient sources, motility styles (nonmotile, motile, various swimming mechanisms), or even structures for organisms that are larger than a few microns.

IV.B. *How* to search for and analyze data in different environments?

B.1. How can we look for extant life in an ocean world remotely (from orbit or during a flyby)?
B.2. How can we look for extant life in an ocean world in *in situ* (landed, underwater, plume) investigations?
B.3. How can we look for extant life in an ocean world with sample return science?
B.4. Which science operational strategies should be used to detect life in ocean worlds?

Although remote detection of extant life seems challenging, the presence of life might be expressed at the surfaces of ocean worlds (B.1). Currently, we do not know what kind of evidence might be seen, at what abundances, or how this evidence might be modified by radiation processing and oxidation. It would also be useful to determine to what extent remote spectroscopy techniques could measure co-located electron donors (*e.g.*, H_2_ ± CH_4_) and acceptors (*e.g.*, O_2_, nitrate, Fe^3+^, CO_2_), and to what extent the geological context or other indicators might allow the inference of how long this co-location has persisted over time. In airless worlds, measuring remotely the surface distribution of elements and looking for any deviations from background bulk concentrations could represent another possible avenue, but relevant spatial scales have yet to be constrained. As a final example, remote spectroscopy techniques could detect pigments and/or other specific biomolecules.

Possible *in situ* investigations for life (B.2) could seek to determine whether the inventory of detected organic molecules differs from those expected to be synthesized by abiotic chemistry. One could look for morphological signatures that indicate microenvironments containing chemical gradients; active, sharp physicochemical gradients of metabolic interest in ice or water columns (*e.g.*, gradients in pH, Eh, temperature); evidence for enzymatic catalysis in the formation pathways of detected organic molecules (*e.g.*, stable isotopic fractionations of CHNOPS that differ from those of abiotic systems over the full range of plausible habitat temperatures); or chemical cycling (organic or inorganic) or electrochemical/electrical activity not explained by abiotic processes that could be indicative of metabolic activity. Planned *in situ* investigations could benefit from lessons learned from previous life-detection experiments, such as the Viking suite. *In situ* investigations can also search for extant, dormant, or extinct life preserved in near-surface ice that results from upwelling through cracks; viable microorganisms have been found preserved in ancient ice on the Earth.

Searches for extant life *in situ* should also include surveys with optical microscopy—under the temperatures and chemistry conditions of environments where extant life is likely to form (liquid water environments), the sizes of organisms will be constrained by the same effects as on the Earth: Chemical diffusion rates will set the upper limit, and the minimum amount of chemistry (information molecules and supporting chemistry) required for self-replication will set the lower limit. Fluorescent dyes that are specific to various classes of molecules without being single-molecule specific can be used to determine whether cell-like objects contain likely biotic chemistry, such as lipids, proteins, amino acids, and sugars. Various stimuli (chemical, photo, magneto, thermo) can be applied to samples to induce changes in activity levels, such as growth, reproduction, or motility.

Possible investigations for life in returned samples (B.3) include determining (1) the distribution of detected organics, and whether it differs from that expected to be synthesized by abiotic chemistry; (2) signatures in the conformation (*e.g.*, chirality) of specific organics that cannot be obtained abiotically; (3) isotopic compositions of inorganic compounds, as well as of H, C, N, O, and/or S in specific organic compounds, and any measurable deviations from expected abiotic compositions; (4) direct observation of microorganisms; (5) assays of residual elemental, molecular, and isotopic composition for remnants of irradiated (*e.g.*, Europan) biological materials; (6) knowledge of the naturally abundant inorganic materials from endogenic or exogenic sources; and (7) the possibility of culturing or replication in returned samples. To inform these investigations, *in situ* contextual measurements at the time of collection are essential.

Key considerations for life detection (B.4) include the choice of sampling location and sampled material (*e.g.*, rocks, ice, water, soil, interface zones), limits of detection, contamination control, and meeting planetary protection requirements. Previous searches for life remotely, *in situ*, and in samples on the Earth have taught us that such searches are highly path-dependent: Decisions on which measurements to make depend on the results of previous measurements, and it is difficult to predict *a priori* which measurements will be needed. Identification of relevant sets of complementary observations and possible decision trees for observation types will be important.

Also crucial is the ability to distinguish environments that are prebiotic, host extant life, and postbiotic (harbored now extinct life), to understand the context of any null result. This requires reducing the possibility of false positives due to forward chemical and biological contamination (separately from planetary protection), by characterizing contamination signals and distinguishing them from indigenous signals (Neveu *et al.*, 2018). It also requires constraining the states in which we might find evidence for life (*e.g.*, live, dead, stasis/frozen, fossilized, chemical residue/metabolic waste products). Relevant planetary protection issues include quantifying any exchange (or lack thereof) of biological material between the Earth and ocean worlds, and identifying any synergies between scientific and planetary protection priorities.

### 3.5. Links to the 2013–2022 Planetary Science Decadal Survey and Solar System exploration

The types of ROW investigations (Table 1) and target bodies of interest to an Ocean Worlds Program are included primarily within the Satellites Theme (Chapter 8) of the 2013–2022 Planetary Science Decadal Survey (Squyres *et al.*, 2011). Within that Theme, the goal of determining “What are the processes that result in habitable environments?” most explicitly connects to the ROW objectives. In addition, ocean worlds are clearly a large part of the Habitable Planets cross-cutting science theme, which includes the goal of determining “Beyond Earth, are there contemporary habitats elsewhere in the Solar System with necessary conditions, organic matter, water, energy, and nutrients to sustain life, and do organisms live there now?”

Many of the ROW objectives also tie into geological history-focused questions—such as the Satellites goals of “How did the satellites of the outer Solar System form and evolve?” and “What processes control the present-day behavior of these bodies?”—and the Primitive Bodies goal of “Understanding the role of primitive bodies as building blocks for planets and life.” These connections are often indirectly related (*i.e.*, they require similar measurements even though the driving question differs), as the focus of the Decadal Survey is on understanding each type of target body, versus the higher-level ROW aim to understand how we can best identify and characterize habitable oceans, and ultimately perhaps life, within these bodies. A summary of links between ROW objectives and Decadal Survey questions is given in Table 2.

**Table 2.** Mapping of Decadal Survey Themes to Roadmaps to Ocean Worlds Objectives

		*Relevant ocean worlds objectives*
Decadal survey cross-cutting science theme (Chapter 3)		
Planetary habitats	Beyond the Earth, are there contemporary habitats elsewhere in the Solar System with necessary conditions, organic matter, water, energy, and nutrients to sustain life, and do organisms live there now?	I. A, B II. A, B III. A, B IV. A, B
Decadal Survey satellites science theme (Chapter 8)		
What are the processes that result in habitable environments?	Where are subsurface bodies of liquid water located, and what are their characteristics and histories?	I. A, B II. A
	What are the sources, sinks, and evolution of organic material?	I. C II. B III. B
	What energy sources are available to sustain life?	II. B III. A
	Is there evidence for life on the satellites?	IV. A, B
How did the satellites of the outer Solar System form and evolve?	How are satellite thermal and orbital evolution and internal structure related?	I. A, C III. A
	What is the diversity of geological activity and how has it changed over time?	I. B
What processes control the present-day behavior of these bodies?	How do endogenic processes shape the satellites' surfaces and influence their interiors?	I. A, B
	What processes control the chemistry and dynamics of satellite atmospheres?	I. B II. B
	How do exogenic processes modify these bodies?	III. B
Decadal Survey small bodies/KBOs science theme (Chapter 4)		
Understanding the role of primitive bodies as building blocks for planets and life	Composition, origin, and primordial distribution of volatiles and organic matter in the Solar System	III. A, B

KBO = Kuiper Belt object.

### 3.6. Links to broader outer Solar System research objectives

ROW-related investigations have close links with science goals pertaining to mission targets throughout the outer Solar System. Identifying ocean worlds and assessing their habitability will be enabled by detailed investigations of many targets even if they do not directly possess oceans themselves. For example, Io is a laboratory for understanding tidal heating, a process that is critical to sustaining ocean worlds. Although Io is not an “ocean world” in the sense used here (despite its potential internal magma ocean), careful application of the lessons learned from studying Io's thermal inventory will prove invaluable in the pursuit of science objectives described throughout this article. Similarly, Ceres is believed to have hosted a global ocean in its early history; this ocean would have frozen within the first few 100 million years of Ceres' evolution, unless convective mixing in a muddy interior slowed down heat loss. A briny layer mixed with silicates could remain at present at the interface between Ceres' crust and mantle, consistent with thermal modeling. Likewise, a more complete understanding of the interiors of the ice giants (*e.g.*, from a dedicated mission) is critical to understanding the evolution of potential ocean worlds around these planets (*e.g.*, Triton, Ariel, Miranda). Specifically, the tidal dissipation factor (Q) of a planet is critical to driving the dynamics and heating in these systems. This factor is a complex function of the interior structure of the planet. The more we understand about ice giant interiors, the more we can learn about potential heat sources, to sustain oceans within the moons.

ROW investigations are also well correlated to the overarching research goals of NASA's PSD. Questions addressed under Goals I and II (Table 1) directly correspond to the goals of NASA's Solar System Workings (SSW) program as they require consideration and investigation of the interior structures, orbital evolution, and the resulting potential surface modification of particular ocean worlds. In addition, Goals III and IV correspond directly to the goals of NASA's HW program, which include assessing the astrobiological potential of ocean worlds, and to the Exobiology program, which places an emphasis on biosignatures and life elsewhere. Further, since ROW's target bodies may potentially serve as analogues for water-rich, habitable exoplanets and exomoons (Léger *et al.*, 2004; Ehrenreich and Cassan, 2007; Tajika, 2008; Fu *et al.*, 2010; Hu and Yang, 2014; Vance *et al.*, 2015), all ROW investigations will also map directly to the research goals of NASA's Astrophysics Division, particularly in relation to the identification and characterization of “habitable exoplanets and/or their moons.” The ROW investigations are also applicable to “understanding the chemical and physical processes of exoplanets, including the state and evolution of their surfaces, interiors, and atmospheres,” which is a primary goal of the Exoplanets Research Program.

## 4. Roadmap to Ocean Worlds

Here, the ROW team outlines a roadmap of initial suites of missions that advance all ROW objectives as outlined in Table 1, with follow-on plans to any one body entirely dependent on what is found during the initial missions. In other words, here we do not plan for contingencies but rather focus on the important next missions to send to different bodies in the ocean worlds spectrum, along with the needed technologies for development. It is assumed that, if a candidate ocean world moves to the category of a confirmed ocean world, this roadmap would be updated and steps would then be taken to characterize that ocean, its habitability, etc. We focus here on priorities that can potentially be addressed in the next decade.

Search-for-life missions should take place at target bodies that are the most likely to support life and should include science payloads that can yield important information (such as a broader context of the sample environment, characterization of prebiotic chemistry as an indication of how far toward life the conditions have progressed, or assessment of the habitability of the environment) even if life signatures are ambiguous or absent in that particular mission. If hints of biosignatures are found, an appropriate follow-on mission should be planned.

In this roadmap are the confirmed ocean worlds Enceladus, Titan, and Europa, which are the highest priority bodies to target in the near term to address ROW goals. Triton is the highest priority candidate ocean world to target in the near term.

### 4.1. Confirmed ocean worlds

Europa, Titan, and Enceladus are confirmed ocean worlds and each is a compelling target in different ways. As known ocean worlds, the next step on the ROW goals list (Table 1) for these bodies is to characterize habitability (as needed) and then, when/if habitability is deemed adequate for life, to search for life.

Ganymede and Callisto are also known ocean worlds, which are of lower priority in the roadmap in terms of characterizing habitability or searching for life. Because these oceans are deeper and there is no evidence of communication between liquid water and the surface and/or a silicate core, oceans at Ganymede and Callisto should be better understood before exploring them as potentially habitable. This lack of knowledge limits their ability to support more of the Ocean Worlds science objectives, and, thus, they are lower in priority from the other known ocean worlds.

#### 4.1.1. Known ocean worlds: target summary and recommendations

##### 4.1.1.1. Enceladus

**FIG. 6.**
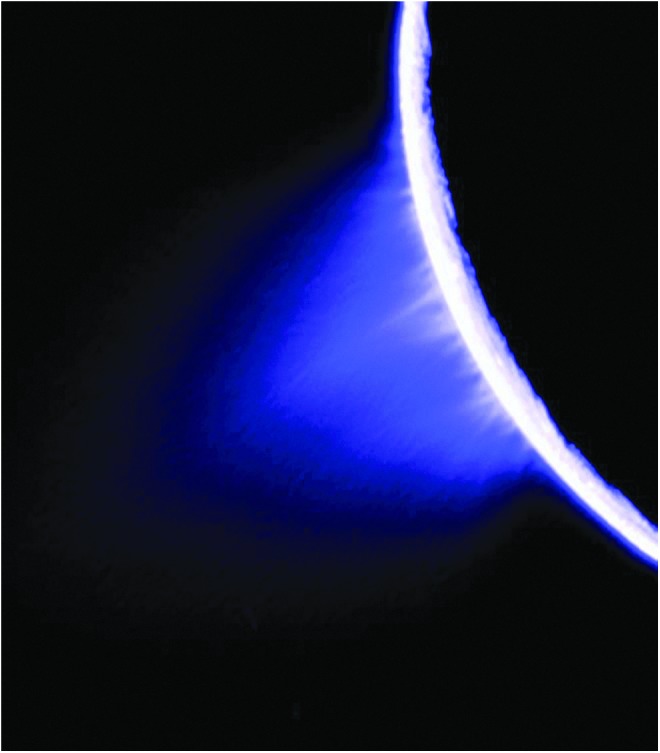
The Enceladus plume, sourced by a potentially habitable subsurface ocean. Credit: NASA/JPL/Space Science Institute.

The habitability of Enceladus' ocean has been sufficiently established by using *Cassini* measurements (Fig. 6), and thus to address ROW goals, a search-for-life mission could be sent as a next step. The ROW team strongly recommends that a search-for-life mission at Enceladus be of high priority in the next decade. Enceladus mission architectures that address the search for life should be studied in advance of the next Decadal Survey. New technologies may need development in addition to that funded for the New Frontiers 4 ELSAH concept.

##### 4.1.1.2. Europa

Europa Clipper is a flagship mission in Phase B of development; the overarching goal of Clipper is to establish the habitability of Europa. An astrobiology-focused Europa Lander mission has been studied (Hand *et al.*, 2017). The ROW team recommends that the Europa Clipper mission continue as planned for its importance in characterizing the habitability of Europa. The ROW team supports a Europa-landed search-for-life mission, especially if the science payload can yield important astrobiological information even if biosignature results are ambiguous. Such a mission will advance the technologies needed to detect biosignatures at ocean world targets, especially from *in situ* measurements.

##### 4.1.1.3. Titan

The habitability of Titan's subsurface ocean and any interfaces between the ocean and surface, along with the surface lakes and seas of methane/ethane, has yet to be established. Thus, a habitability/ocean characterization mission to Titan is a natural next step to advance ROW goals at this body. Numerous types of missions at Titan are possible. The Dragonfly mission concept has been selected for a Phase A study for New Frontiers 4. The ROW team considers missions to characterize Titan's ocean and assess its habitability to be of high priority. Even if Dragonfly is selected for New Frontiers 4, additional Titan missions that advance the understanding of Titan as an ocean world should be studied before the Decadal Survey and considered by the Decadal Survey panel.

##### 4.1.1.4. Ganymede

The ESA JUICE mission is set to explore Ganymede. This mission will characterize Ganymede's subsurface ocean, located between layers of near-surface and high-pressure ices, to better understand the formation and evolution of this ocean world. It could place bounds on communication between the subsurface ocean and the surface, energy input into the ocean layer, and the habitability of oceans separated from underlying rocky mantles. The ROW team supports the ESA JUICE mission.

##### 4.1.1.5. Callisto

This known ocean world remains to be fully characterized. Its deep subsurface ocean and its location on the edge of the Galilean satellite system limits not only communication between the ocean and the surface but also vital energy input to the ocean. It may serve as an end member on the ocean world spectrum and help, along with Ceres, to characterize the limit of the ability of bodies to maintain oceans with sparse tidal input. In addition, because Callisto's ocean is also located between two layers of ices, Callisto studies could inform studies of Ganymede's ocean. The ROW team supports mission studies to characterize Callisto's ocean and its sustainability. A smallsat mission to Callisto should be studied, which can perhaps advance ROW objectives.

### 4.2. Candidate ocean worlds

Triton, Pluto, Ariel, Miranda, and Ceres are among the possible ocean worlds in the Solar System. Spacecraft data returned from these bodies suggest the possible presence of extant liquids in their interiors, but the size of any liquid reservoir is unknown. These bodies must be explored further to determine whether they have extant oceans and whether they should be further studied as ocean worlds. The next missions to these bodies should establish the presence of oceans, perhaps using orbiting spacecraft (or multiple flyby missions) with magnetic, gravity field, libration, and/or topographic measurements of tidal flexing. Should extant oceans be found, future missions should characterize those oceans to establish their habitability and then potentially search for life.

In ranking the priority of the worlds just described, we consider two factors: the timing of geological activity, suggesting the presence of an ocean, and the likelihood of this activity being endogenic (including tidal) as opposed to exogenic (driven by insolation or impacts).

Other possible ocean worlds exist. However, the bodies listed here represent the most likely targets on which we can confirm oceans in the near future.

#### 4.2.1. Candidate ocean worlds: target summary and recommendations

##### 4.2.1.1. Triton

**FIG. 7.**
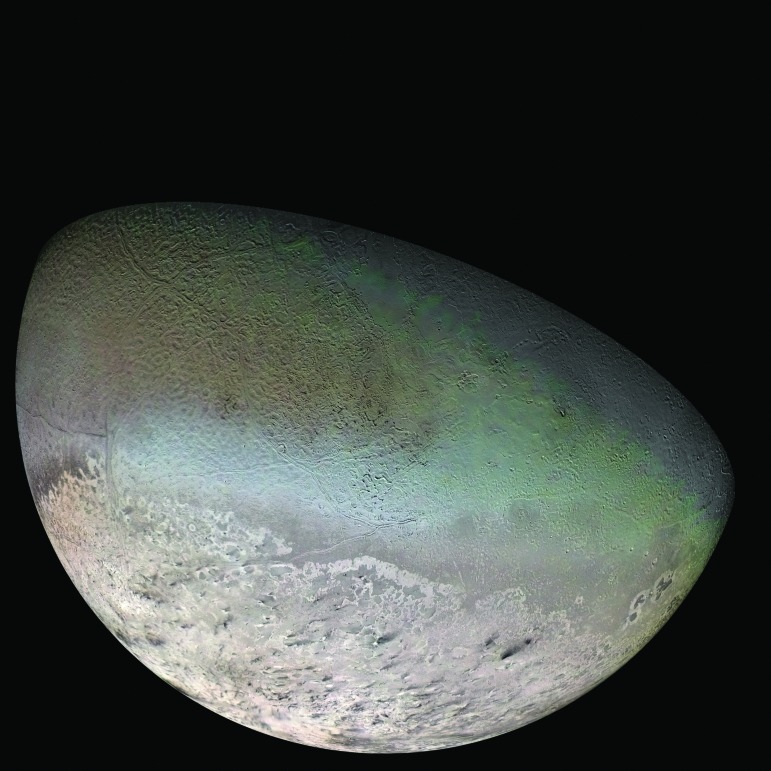
Voyager 2 image of Neptune's moon Triton. Image credit: NASA.

Of the worlds just cited, Triton is deemed the highest priority target to address as part of an Ocean Worlds Program. This priority is given based on the extraordinary hints of activity shown by the Voyager spacecraft (*e.g.*, plume activity; smooth, walled plains units; the cantaloupe terrain suggestive of convection) (Fig. 7) and the potential for ocean-driven activity given by *Cassini* results at Enceladus. Although the source of energy for Triton's activity remains unclear, all active bodies in the Solar System are driven by endogenic heat sources, and Triton's activity coupled with the young surface age makes investigation of an endogenic source important. Further, many Triton mission architectures would simultaneously address Ice Giant goals on which high priority was placed in the Visions and Voyages Decadal Survey. Finally, as Triton likely represents a captured Kuiper Belt object (KBO), some types of comparative planetology with KBOs could also be addressed in a Triton mission. Before the next Decadal Survey, a mission study should be performed that would address Triton as a potential ocean world; such a study could be part of a larger Neptune orbiter mission. The Decadal Survey should place high priority on Triton as a target in the Ocean Worlds Program.

##### 4.2.1.2. Pluto

Pluto is the first large object visited in the Kuiper belt and it shows young, potentially cryovolcanic terrains, indicating activity may have continued through much of its history. As for Triton, the source of relatively recent internal heat on Pluto is not well understood, but models suggest that an ocean may persist into the present. Studying large KBOs opens up a new regime for exploring ocean worlds in the Solar System, and by comparative planetology helps us understand what is possible for icy moons that are not currently tidally heated. Mission studies should be performed to address technology advances enhancing exploration of the Kuiper belt or a return to Pluto with an orbiter (necessary to study a potential ocean). Studies to explore a potential KBO rendezvous as an extended part of another mission to the outer Solar System (*e.g.*, to a gas giant) are also encouraged.

##### 4.2.1.3. Ariel and Miranda

After the Voyager flyby of the outer Solar System, similarities between Enceladus, Miranda, and Ariel were noted. Only after *Cassini's* arrival were Enceladus' extant geological activity and ocean discovered. Both Miranda and Ariel show evidence for recent significant tectonism that could indicate subsurface oceans. A mission to the Uranian system should set, as a top priority, flybys of these moons to search for evidence of subsurface oceans.

##### 4.2.1.4. Ceres

Ceres is a unique case, a hydrous dwarf planet in the asteroid belt. Ceres is ∼50% H_2_O in volume and has a 40-km-thick shell dominated by volatiles, with a density of 1.28 g/cm^3^, but whether there is liquid water in its interior today is the subject of ongoing analyses of data from the *Dawn* spacecraft. Ceres is a small and heat-limited body, likely in the process of freezing, so it may provide an end-member scenario for medium-sized ocean worlds without tidal heating. Modeling and experimental research (utilizing Research and Analysis [R&A] funding) in light of *Dawn* results would inform the understanding of ocean worlds as a whole. A Ceres mission with a primary objective to detect and characterize any liquids within Ceres should be studied to determine how well small mission classes can help advance ROW objectives.

## 5. Mission Scenarios

### 5.1. Enceladus

The next steps for exploration of Enceladus should build on the science achievements of the *Cassini* mission and leverage existing and developing technologies to best assess the habitability or presence of biosignatures (extinct or extant) on this moon. The following sequence of missions is proposed, but not explicitly required, to achieve these objectives:

1. Plume flyby (*in situ* analysis)—to search for complex organics indicative of life; and to identify the best way to capture and preserve a sample for sample return.
2. Plume flyby+sample return—to enable higher fidelity analysis by utilizing the latest laboratory techniques and instrumentation on the Earth to search for evidence of life in the plume. An alternative approach would be to use a Lander to access material for sample return. This would enable a much larger sample size to be collected, and it would not rely on plumes being active for the sample return mission.
3. Lander—this mission would look for markers (and potentially cells) in the larger, heavier plume fallout grains on the surface that cannot be safely accessed in a flyby. An option could be to combine this with a “crawler” (below).
4. “Crawler” to access deeper into the Tiger Stripes (possibly to the ocean)—to test new technologies for accessing chasms of similar ocean worlds without drilling/melting.
5. Submarine to access the ocean (delivered via either “crawler” or melt probe)—in this final phase, the ocean of Enceladus would be sampled directly in the search for microbial activity or other evidence of life. Depending on design, a submersible or something similar might reach the ocean floor to verify the presence of, and collect samples from, hydrothermal vents.

We note that any mission architecture must remain flexible to accommodate discovery-driven changes. From Sherwood (2016): “Among the many “ocean worlds” of our Solar System, Enceladus appears unique in its combination of astrobiologically relevant and exploration-worthy attributes: extensive liquid-water ocean with active hydrothermal activity, containing salts and organics expressed predictably into space. The Enceladus south polar plume allows direct access to telltale molecules, ions, isotopes, and potential cytofragments in space. Plume mass spectroscopy and sample return, *in situ* investigation of surface fallback deposits, direct vent exploration, and eventually oceanographic exploration can all be envisioned. However, building consensus to fund such ambitious exploration hinges on acquiring key new data. A roadmap is essential. It could start with cost-capped onramps such as flythrough analysis of the plume, following up on *Cassini* measurements with modern instruments; and sample return of plume material for analysis on Earth. A methodical mission sequence in which each step depends on emergent results from prior missions would push *in situ* oceanographic exploration into the second half of this century. Even for this scenario, prioritization by the next planetary Decadal Survey would be pivotal.”

Thanks to the pioneering work of *Cassini*, the first major steps in the astrobiological exploration of Enceladus have already been accomplished: (1) A global, subsurface ocean was identified and confirmed; (2) simple and complex organics have been detected in a plume that is sourced from the ocean; (3) multiple independent lines of evidence strongly suggest hydrothermal activity; and (4) the optimal landing region for a Lander has been established (the Tiger Stripes); further high-resolution reconnaissance is needed to find a safe landing site within the plume fallout zone. This puts Enceladus in a pivotal role as one of the pathfinding targets where we may test our mission strategy to explore ocean worlds. The optimal strategy must minimize the number and scope (and therefore cost) of missions needed to address the question (is there life on Enceladus?) while still achieving the scientific rigor needed to test such a momentous hypothesis.

One unique issue for an Enceladus astrobiology mission is that nearly all orbital trajectories to target this small moon must also include a Titan flyby. Instruments designed for ultrasensitive detection of organics must be adequately isolated from contamination during this Titan encounter; this may be a nonissue given that a flyby well outside of Titan's atmosphere can be used for this purpose. Inlet covers and/or decontamination heaters may help to address this concern.

### 5.2. Europa

The next mission to Europa follows up on the *Galileo* mission with a comprehensive remote-sensing survey, including global mapping, magnetometry, and radar to sound the subsurface to attempt to confirm the presence of and depth to a subsurface ocean. Such a mission is currently under construction at NASA, and the Europa *Clipper* mission will extensively characterize Europa's surface. Europa *Clipper* includes a radar sounding instrument and magnetometer to probe the subsurface structure of Europa, and *in situ* instruments to measure the composition of particles in Europa's exosphere, lofted into space by impacts, sputtering, and/or plumes, if they exist.

One science objective not well addressed by *Clipper* is gravity science. A low-altitude orbiter mission such as *GRAIL* can revolutionize our understanding of Europa, and it may be achievable within the Discovery or New Frontiers program.

The next Europa Flagship mission after the global reconnaissance mission should be a Lander. Such a mission would be able to not only search directly for life on another planetary body but also provide valuable information on the geological nature of the surface. Much can be done remotely to characterize Europa's habitability, but likely a true search for life will come from a landed mission that directly samples Europa's near-surface material to search for signs of life. Such a Lander mission would sample the subsurface and perform visual (microscopic) and compositional (GCMS or other instrument) analysis of materials that come from Europa's near-surface.

This first Europa Lander would likely choose a landing site based on compositional heterogeneity as well as safety. Likely the highest priority landing site would be one where subsurface material has made recent (very recent, geologically) contact with the surface. Such a landing site would likely be selected based on a complete orbital survey of the surface, to look for compositional indicators of recent activity (such as plume deposits), regions where communication between the surface and subsurface are likely to occur, as evidenced by high concentrations of nonice materials (Shirley *et al.*, 2010), and/or regions in the ice shell where geological processes such as subsumption (Katterhorn and Prockter, 2014) and ridge formation (Greenberg, 2010), among others, may facilitate the cycling of surface species into the subsurface. As warm ice diapirs may be habitable, and their movement through the crust will heat the surrounding country ice (Quick and Marsh, 2015), producing transient habitable environments that may last 10^4^–10^5^ years (Ruiz *et al.*, 2007), chaos regions, and lenticulae fields could also be prime targets for subsurface sampling. Engineering constraints would also come into play, as we consider what kind of surface a lander could successfully access.

A Europa Lander mission concept is currently under study by NASA. Follow-on missions would likely depend on the results of the first Europa Lander mission, and whether it detects clear biosignatures. Such future missions would likely include more capable landers, rovers, and eventually a submersible or melt probe that would directly access the subsurface liquid ocean layer and might even return samples (Sherwood, 2016). Seismology on a lander would greatly facilitate future exploration into Europa's ice shell or ocean. A Lander relay orbiter could include enhanced capabilities to add to *Clipper* science (*e.g.*, geodesy).

Innovative concepts for Europa exploration should be encouraged in Discovery and New Frontiers.

### 5.3. Titan

Titan is a diverse and multifaceted world that is arguably more complex than the other ocean worlds. Multiple missions will be needed to reveal Titan and how it fits in with other Solar System bodies.

The complex photochemical synthesis of Titan's methane-rich atmosphere leads to deposition of a wide variety of organic molecules, an active weather system, and fluvial and aeolian geological processes. The potential for exchange and contact of these organics with water creates the potential for chemically rich environments in the deep subsurface ocean. At the surface, transient liquid water environments could exist after impact events and possible cryovolcanic eruptions, providing a short-lived habitat for life. The hydrocarbon seas and lakes may also serve as regions where prebiotic chemical processes could potentially evolve into an organized biochemistry. If these organics reach the water ocean, then this world would be especially interesting.

Future missions are needed to determine the origin of the atmosphere, the length of time the photochemical production cycle has been running, the complex organic synthesis of the upper atmosphere, the properties of the hydrocarbon seas, geodesy and geomorphology of the surface, chemical sampling of the plains, dunes, water-ice rich mountains, targeted sampling of locations where water has modified surface organics (*e.g.*, impact melts), and perhaps even locations where the subsurface ocean has broached the surface (*e.g.*, cryovolcanic flows).

Multiple mission concepts are needed to fully explore Titan. Next, we list a variety of these concepts. Each concept could be proposed as a single mission (*e.g.*, the TiME lake lander proposed to Discovery 12), or multiple concepts can be combined into more ambitious mission proposals (*e.g.*, the Oceanus orbiter proposed to New Frontiers 4, which combines items 1, 2, and 3). It is also relevant to note that the Saturn system contains another high-priority ROW target, Enceladus. Mission concepts that combine elements of the list given next along with elements of the Enceladus exploration strategy (see Sec. 5.1) should be considered (*e.g.*, the Journey to Enceladus and Titan Saturn orbiter proposed to Discovery 12).

1. Titan orbiter: This concept would include high-resolution imaging, topographical grid, atmospheric chemistry, and weather monitoring from orbit. Mapping could be from IR imaging or SAR radar. Topography from radar or laser altimetry would determine the tidal Love numbers and provide constraints on the thickness of the ice shell and interior structure (including information that will help determine whether the interface with the rocky interior is liquid or high-pressure ice). The mission could include a search for near-surface water pockets and/or subsurface hydrocarbon reservoirs (may require a REASON style deep radar instrument). It could also include initial instrumentation to examine chemical synthesis in Titan's upper atmosphere. For longer duration orbital missions, weather processes could also be tracked. It could include synoptic observation, microwave radiometer mapping, and monitoring of haze structure with ultra-violet (UV).
2. Lake lander: This mission concept would focus on sampling of lakes (*e.g.*, TiME). A short-lived mission would provide determination of bulk composition, trace dissolved species, and sediment depths of lakes (sonar, radar). A longer duration mission could provide information of winds, currents, and tides. A lake lander can be included as an additional element in an orbiter or flyby concept.
3. Titan atmospheric probe: This probe would carry instrumentation that would be able to examine polymeric materials made in the upper atmosphere, and examine how concentrations, functionalities, and structures vary with depth from the exobase (1450 km) to the condensation point (150 km).
4. Aerial mission: A Titan airborne platform could explore across multiple terrain types (*e.g.*, Titan Aerial Explorer study or AVIATOR or Dragonfly mission concepts), could study geomorphology, and do terrain classification at 1-m resolution or better. A key component of this concept is the ability to sample multiple terrain types with either *in situ* spectral capability (*e.g.*, UV-Raman) or GCxMS. A Titan airplane or helicopter would allow directed navigation to different terrain types. Directed targets could include potential cryovolcanic areas, lakebeds, impact craters, dunes, and plains. Similar to the lake lander, a balloon can be delivered by an instrumented orbiter or a flyby element.
5. Submarine: This concept would provide *in situ* exploration of a Titan sea (most likely Kraken Mare). The submarine would measure the composition of the liquid (with depth), investigate surface and subsurface currents, look for mixing and layering, observe wind and waves at the surface, map sea bathymetry and bottom features, and determine the seabed sediment composition. Such a mission would require a significant amount of autonomy, and it could either be designed as a Direct to Earth concept or make use of an orbital relay.
6. Drill for deep exploration of subsurface ocean *in situ*: This concept will need to fully understand the physical properties of Titan's organic inventory. Thus, it will require other landed elements (such as those described earlier) to have returned data first. The initial drill could target cryovolcanic areas, or if found, shallow water diapirs or deep subsurface hydrocarbon layers.

### 5.4. Triton

The next step at Triton is to send a mission with the following two objectives:

1. Determine whether Triton has an internal ocean. This could be accomplished by looking for the magnetic induction signature and/or gravity field measurements and/or searching for libration with high-resolution images and/or LIDAR.
2. Determine whether Triton's plumes sample a subsurface liquid layer. High-resolution images to look for ongoing eruptions are the minimal data set, but additional information on composition is important. The composition of the plumes could be assessed by using UV spectroscopic imaging of solar occultations from a spacecraft at the Neptune-Triton system. The UV spectra can be used to derive density profiles of different species in the atmosphere. If the solar occultation probed a plume, then we would have direct information about the composition of the plume and therefore the subsurface source. Mass spectrometry could also be useful.

There is tremendous additional science to be achieved by mapping surface volatile distribution, understanding the global geology and surface unit ages, etc. so the two objectives just represent a minimum threshold.

In addition to the technologies that will benefit exploration of all ocean worlds (*e.g.*, improved power systems such as eMMRTGs and larger launch vehicles such as the SLS), a Triton mission would benefit greatly from the ability to aerocapture at Neptune. It is worth noting, however, that great science is also possible with a Neptune-Triton flyby. Description of such missions already exist and rather than repeat that work here, we refer readers to the Argo white paper (Hansen *et al.*, 2009) and the Ice Giants SDT report (Hofstadter *et al.*, 2017).

Similar to KBO and Pluto studies, Triton investigations will benefit from improved understanding of the rheology of N_2_ ice and water-ice mixtures (water-ammonia, methane clathrates) at the temperatures expected on these targets. In addition, lab work to understand the spectral signatures of mixtures of ices in solid solution would be beneficial as well. Specifically, phase diagrams for mixtures of species such as N_2_, CH_4_, CO, and CO_2_ are needed as well as optical constants in the UV and IR.

### 5.5. Ganymede and Callisto

A Ganymede mission was studied for the Visions and Voyages Decadal Survey and that study is still relevant. The JUICE mission may cover many of the goals of that concept study. Beyond that, the Jupiter System Observer (JSO) concept study included a Ganymede-orbiting architecture. JUICE essentially accomplishes many of the ROW-related goals of JSO; JUICE is currently lacking mainly the Jupiter and Io observing aspects, which do not fall under the ROW umbrella (although both are relevant to understanding ocean worlds, such as tidal heating).

Since JSO implemented a Ganymede orbiter, it might be worth reviving this “staying out of the system” option, where the focus of the mission is at Ganymede and Callisto. Neither JUICE nor Europa Clipper is going to do much science at Callisto, and JUICE's science goals for Ganymede are going to be impacted by the fact that they may not have the Δv to get into the 200-km circular orbit they originally planned for.

### 5.6. Ceres and small bodies

Here, we focus on Ceres since it is the only asteroid that offers any prospect for being an ocean world at present, both from theoretical considerations and from observations returned by the *Dawn* mission.

The next step in the exploration of Ceres is to develop observational strategies to approach the following objectives:

1. Characterize the extent of the deep liquid layer inferred from models of Ceres' relaxation and characterize Ceres' thermal state.
2. Assess the habitability of Ceres' past and current ocean via the determination of its physicochemical properties.
3. Confirm the origin of organic material found in the Ernutet region and characterize the dark component throughout the surface, which is believed to be evolved from carbon species; determine the conditions under which these carbon-rich materials were formed.

In-depth studies are needed to assess which mission architecture and payload are most suitable for this unique body, rich in salts and volatiles but with putative endogenic activity limited to only a few places. For example, one needs to identify which observational strategy is best suited when searching for a deep ocean (Objective 1) in a body not subject to tidal forcing.

Objectives 2 and 3 require *in situ* exploration and would likely leverage instrumentation and operations developed for the Mars and Europa landers. Ceres is an airless body located at ∼2.7 AU from the Sun, with surface temperatures up to ∼240 K at the equator but ∼170 K on average. These temperatures will impact mission lifetime and operational strategy, especially for missions at high latitudes (*e.g.*, the Ernutet region is located at about 55° latitude), and might call for radioisotope power sources.

Answers to the objectives just cited will determine whether follow-on exploration is warranted to address ROW *Goal IV'*s “how life might exist at each ocean world and search for life.”

Planetary Protection: Another important question to be addressed for future exploration of Ceres is under what Planetary Protection Category these missions would fall. Crater counting indicates that most of Ceres' surface is older than 100 My except in a few places that display geological evidence for recent or perhaps ongoing communication between the interior and the surface. Ahuna Mons is interpreted as a cryovolcano that has been dated to be as young as 70 Ma. However, its age may be revised as our understanding of crater-based chronology improves. The depth of Ahuna's source is unknown.
Occator Crater is <25 Ma but the bright salt deposits found on its floor could be only a few megayears old or even being exposed at present. Whether the source material comes from a melt reservoir created by impact-produced heat and/or communicates directly with the deeper brine reservoir is unknown. To avoid impact with the Occator faculae, the *Dawn* spacecraft was required to remain on a 20-year stable end of mission orbit. Planetary protection technologies developed for Mars or Europa may be applicable to a future landed mission, if it targets the Occator faculae and potentially Ahuna Mons.
Ceres as a Stepping Stone for the Exploration of Ocean Worlds: Ceres represents an important data point for understanding the chemical evolution of volatile-rich worlds and especially their potential for forming and preserving organic compounds. With its low gravity and relative benign environment, Ceres also offers easy surface access (in comparison to Mars or Europa) whereas the roundtrip light-time to/from Ceres requires the introduction of semi-autonomous techniques for advanced surface operations. Hence, a long-term exploration program of Ceres is compelling, not just for the anticipated science return but also because it will help us practice and hone new technologies of relevance to the future exploration of ocean worlds, such as surface operations, planetary protection, and end-to-end sample collection and return to the Earth.

### 5.7. Pluto/KBOs

Visiting new bodies in the distant outer Solar System as well as a return to Pluto/Charon would be extremely informative for understanding the evolution of our Solar System. As such, it is informative to visit as many large KBOs as possible, for the following reasons:

- Large, bright KBOs likely have the best chance of being ocean worlds. The large size allows for more internal heat and the bright surface may be the result of geologically recent, or even ongoing, activity. Higher density KBOs (only known if they have a companion) have more potential for radiogenic heating and are more likely to be differentiated. The magnitude of tidal heating is unknown for multiple-KBO systems; thus, visiting multiple-body systems, even if they consist of smaller objects, will be important data points.
- We do not currently have enough constraints on composition for KBOs (*e.g.*, What makes up all the red material observed on KBOs? How much variety exists in KBO bulk interior structures? What is the distribution of bio-essential elements?). Any missions/instrumentation that bear on such questions would be prioritized for an ocean worlds focused mission.
- Presumably orbiting any of these objects is technologically prohibitive, but even flyby missions would provide a wealth of information. Some of the composition information could even be better sampled by a lander if such a mission were possible in the future.

A return to Pluto/Charon is needed to reveal whether Pluto has a present-day ocean (a possibility given some of the young surface terrains and also an outcome produced by some models of interior evolution), and to characterize Charon's past ocean. An orbiter would significantly advance Pluto/Charon science and is likely needed for true characterization of the ocean (*e.g.*, via gravity science); however, an additional flyby would also be useful. Many unanswered questions remain about this double dwarf-planet system, and New Horizons observed only ∼40% of the surface area each of Pluto and Charon at moderate-to-high resolution. The Pluto system provides an opportunity to study a two-body system where one world may have retained an ocean into the present, whereas the other world's ocean froze. Further, the Pluto system is also unique in that it is not tidally forced today, and thus a present-day ocean in Pluto would represent a different kind of interior evolution than those of the giant-planet satellites.

Research into ways to optimize distant outer Solar System missions is needed. For example:

- Are there opportunities to piggy-back on other missions to the outer Solar System? (*e.g.*, giant planet missions)?
- Can multiple large KBOs be visited with a single spacecraft?
- Can multiple smaller/cheaper spacecraft be sent to visit different KBOs?
- Are KBO orbiters possible with current technology and within cost constraints?
- Are KBO landers possible with current technology and within cost constraints?
- What opportunities do different launch vehicles and power sources enable?

### 5.8. Other satellites

#### 5.8.1. Saturnian satellites (Mimas, Tethys, Dione, Rhea, Iapetus)

Some of Saturn's medium-sized satellites have had gravity science flybys by *Cassini*, whereas others have not had any close flybys. In all cases, this class of satellite would greatly benefit from multiple close flybys that are dedicated to gravity science. Iapetus has only had one relatively close flyby by *Cassini*. In addition, the innermost mid-sized satellite Mimas has had no dedicated flybys. For ocean detection, future missions should focus on gravity and high-resolution imaging to measure librations (Tajeddine *et al.*, 2014; Thomas *et al.*, 2016), similar to what has been done at Enceladus (Iess *et al.*, 2014; McKinnon 2015; Thomas *et al.*, 2016), especially at Dione.

#### 5.8.2. Uranian satellites (Miranda, Ariel, Umbriel, Titania, Oberon)

At Uranus, a flyby mission to the whole system to image the regions of the satellites that were in darkness during the Voyager encounter would greatly enhance our knowledge of the system. However, a *Cassini*-style orbiter at Uranus would yield the greatest benefit for the system (Hofstadter *et al.*, 2017). Such a mission should be outfitted with instruments that would be useful for analyzing the parent planet, rings, and the satellites. For example, a magnetometer and a high-resolution imaging camera would be key. For the purpose of gravity science and electromagnetic sounding, it would be necessary to perform multiple close flybys of each moon. Ideally, a mission to Uranus would occur at equinox (2049) or northern summer (2028) to enable imaging of the hemispheres that were not visible during the Voyager flybys. Alternatively, having instruments that can analyze the dark portions of the satellites would help fill in any gaps, that is, laser altimeter, active radar, and/or passive instruments that are sensitive enough to use “Uranus shine” or thermal emission to image the shadowed surface.

## 6. Additional Major Findings of the ROW Study

Besides laying out scientific priorities and goals of an Ocean Worlds Program, the ROW group produced two additional major findings in their discussions.

One major finding is that progress needs to be made in the area of collaborations between Earth ocean scientists and extraterrestrial ocean scientists. We can harness the >100 years of ocean research that has been accomplished on the Earth and bring that to bear on future studies that help move the Ocean Worlds Program forward. Stimulation of a program of comparative oceanography will require coordination between agencies. Classical oceanography might not currently fit well within NASA's R&A portfolio; however, the work that the National Science Foundation (NSF) (process studies), the National Oceanic and Atmospheric Administration (NOAA) (exploration), and the Office of Naval Research (ONR) (technology, especially autonomy/robotics) all do in supporting different aspects of ocean research on the Earth is something that they tend to only support under conditions (such as pressure, temperatures, ocean salinity, seafloor composition) that pertain specifically to the Earth. Extending this basis of Earth-centered knowledge into the Solar System will be a challenge and requires a shared vision among the agencies just described. Thus, the ROW team recommends the establishment of a working group to study the specific research areas that can be investigated by direct collaborations between the Earth ocean and the ocean world communities.

A second major finding of this study is that to map out a coherent Ocean Worlds Program, significant input is required from studies here on Earth: Rigorous R&A studies are called for, to enable some future ocean worlds missions to be thoughtfully planned and undertaken. Many research objectives and investigations involve questions that can be addressed here on Earth—through modeling, field studies, lab work, etc. so that spacecraft observations can be best planned, acquired, and interpreted. Most of the ocean world mission candidate bodies are in the outer Solar System, meaning that total mission duration can be decades in length, and fully addressing the open science questions will likely require multiple missions to each body. Given these long timescales, such Earth-based investigations should be undertaken beginning immediately and continue on in parallel with planning and execution of ocean worlds missions. The objectives laid out in this article cover both those that include measurements required to be made at the various target bodies and measurements/studies that will need to be made here on Earth to prepare for those robotic measurements and to help in their interpretation. Thus, the ROW team recommends a rigorous R&A initiative as part of the Ocean Worlds Program; many of these R&A studies could be addressed as part of the current NASA R&A programs, and it is recommended that ocean worlds be highlighted in those programs so that this work can be accomplished. Equally importantly, however, there are other key areas of research that do not fit into current NASA R&A programs (notably, oceanography and ocean interfaces studies).

Basic research is needed in all four goal areas, as described here.

### 6.1. Goal I areas

Objective I.C. (How do materials behave under conditions that are relevant to any particular target body?) and its investigations:

1. What are the phase relations of materials composing ocean worlds at relevant pressures and temperatures?
2. What is the composition and chemical behavior of materials composing ocean worlds?
3. What are the rheologic mechanisms by which material deforms under conditions that are relevant to ocean worlds?
4. How does energy attenuation/dissipation occur under conditions that are relevant to ocean worlds?
5. What are the thermophysical properties of material under conditions that are relevant to ocean worlds?

Many Goal I areas are currently addressed in the Emerging Worlds and SSW Programs. Applications and relevance to ocean worlds should be emphasized in these programs.

### 6.2. Goal II areas

1. New collaborations are needed that interface with Earth-based ocean researchers to pursue comparative oceanography. In the United States, funding for oceanographic process studies is predominantly awarded through the NSF whereas NOAA supports ocean exploration activities. The ONR also supports development of new technologies with—most relevant to this work—an increasing interest in long-range autonomous robotic systems. By design, however, studies conducted through these agencies focus exclusively on conditions that are relevant to the Earth's oceans. (See also the first major finding in Sec. 6)
2. Ice shell-related studies, for example, radar reflectivity
3. Studies are needed that are related to seafloor and subseafloor water/silicate interactions at ranges that are relevant to the pressures, temperatures, and chemical reactants that are relevant to the full spectrum of ocean world settings anticipated (such topics may be covered in HW and SSW); thermodynamics/chemistry of water–rock interactions (also may be appropriate for HW and/or SSW). There has been significant past investment into processes associated with water-rock reactions on the Earth, often done through NSF, but they do not necessarily extend to the full range of conditions anticipated in all ocean worlds.

### 6.3. Goal III areas

“Fundamentals of Habitability” questions:

1. Solvent
1.1. What solvents are suitable for life?
1.1.1. Are there limits (water activity, ionic strength, other) to the composition of aqueous environments that can support life? If so, what are they?
1.1.2. Are solvents other than water capable of supporting life? If so, what are they?
1.2. How do the spatiotemporal extents of liquid environments factor into habitability? How short/transient/small is insufficient? Can a liquid environment be too extensive in space and time to be habitable?
2. Energy
2.1. Can life take advantage of energy sources other than those known to support life on the Earth (redox or visible-near IR light)?
2.2. When and how does energy availability constrain the type, diversity, and/or abundance of life?
2.3. Are there organismal, local, or planetary scale limits on how much energy is enough to support life? If so, what are they, and how do they depend on the physicochemical environment?
2.4. Are there upper limits to the amount of energy that can be constructively harnessed by life (vs. that same energy becoming destructive)?
3. Elemental and molecular raw materials
3.1. Can biochemistry be based on elements other than those utilized by life on the Earth?
3.2. Are there lower limits on the abundance or constraints on the chemical form of elemental and molecular resources required for habitability?
4. Physicochemical environment
4.1. Can life transcend the physicochemical limits exhibited by life on the Earth? Of particular importance for ocean worlds, what are the high-pressure limits for life?
4.2. How do the physicochemical limits for life change under conditions of compound “extremes” or energy limitation?
5. Origin of life
5.1. Under what physicochemical conditions, with what energy sources, and with what abundance and chemical form of elemental and molecular raw materials can life emerge? How does the probability of an origin of life vary within the range of permissive conditions?
5.2. How does the timescale required for life to emerge vary as a function of conditions within the permissive set? What is the absolute value and range of that timescale? (How long must habitable environments be habitable for life to emerge?)
5.3. Are there processes essential to the origin of life that definitively do not occur in sub-ice oceans?

Many of these Goal III questions are covered in the Exobiology program. Applications and relevance to ocean worlds should be emphasized in this program.

Note that some of these are extremely challenging questions that we cannot reasonably expect to answer in any short-term horizon, for example the question relating to “the probability” of the origin of life. The purpose in articulating them here is to create a complete list of open questions whose answers could strongly influence the way we choose to (prioritize a) search for life, whether or not we think those answers will be forthcoming in a meaningful time frame.

### 6.4. Goal IV areas

1. Biomarkers
1.1. What biomarkers best combine unambiguity of feature and interpretation, nonspecificity to Earth-like life, low likelihood of false positive or false negative, and ease of detection?
1.2. How do we detect such biomarkers?
1.3. How do biomarkers change under the conditions (*e.g.*, radiation, vacuum) that prevail at the locations at which they can be observed, collected, or otherwise measured? For example, what is the biosignature preservation potential for organics interacting with liquid water in ocean world surfaces?
1.4. How do we get past the disconnect between chemical detection and life (*e.g.*, whole cell, ecosystem) detection and characterization?
2. Biology in environments that are relevant to ocean worlds
2.1. What metabolic strategies are expected to occur in ocean worlds?
2.2. What survival strategies are expected to occur in ocean worlds, and what do these imply for habitable niches on these worlds?
2.3. Are these strategies, or others, applicable to life in solvents other than liquid water; and how?
3. Planetary protection
3.1. To what extent have Earth and ocean worlds exchanged material throughout the history of the Solar System?
3.2. Would an Earth-based biomolecule brought to the surface of an ocean world definitely end up in its ocean? Over what timescales?
3.3. What protocols that are specific to ocean worlds should be implemented for *in situ* and sample return missions, and what are their technological implementations and associated costs?
3.4. What is the appropriateness of the crux planetary protection requirements, including the 1e-4 probability for introducing a viable Earth organism into an alien body of water? This requirement stems from the Viking era, based on hospital sterilization capabilities dating back to the 1940s; is it still appropriate for a 21st-century Ocean Worlds program?
4. Technology
4.1. What biology technologies can be applied to the search for life in ocean worlds?
4.2. How can these technologies be validated and matured?
4.3. Other technology needs for operations in extreme environments

Many Goal IV questions are covered in current R&A lines of funding such as NASA's Habitable Worlds, Exobiology, Astrobiology Institute (NAI) and its working groups (*e.g.*, Biosignatures), Concepts for Ocean worlds Life Detection Technology (COLDTech), and Planetary Protection Research; as well as NSF programs. Applications and relevance to ocean worlds should be emphasized in these programs, as is already the case with COLDTech.

## 7. Suggested Decision Rules and Considerations for the Next Planetary Decadal Survey

1. The next Decadal Survey should rank highly an Enceladus mission (whatever the class).
2. The next Decadal Survey should place an especially high priority on a mission to study life/habitability at Enceladus and/or Titan. A mission that addresses both Enceladus and Titan should be considered (even if the Dragonfly mission is selected for New Frontiers 4).
3. If Europa Lander is in development, or if it is under consideration, by the time of the next Decadal Survey, the panel should recognize the criticality of exploration of additional ocean worlds, thereby placing high priority on missions to Titan, Enceladus, and Triton as well. Technologies developed for Europa Lander should be leveraged for the *in situ* exploration of other ocean worlds.
4. Ice Giant missions under consideration by the Decadal Survey panels should prioritize ocean world science, particularly at Triton, Ariel, and Miranda.

## 8. Summary of Recommendations

The ROW study advocates an Ocean Worlds Program that utilizes different classes of missions (Flagships, New Frontiers, Discovery, and, as possible, smallsats to ride along with these missions) to address ROW questions. These questions focus on (1) understanding where/why oceans are present, which allows for (2) characterizing ocean environments in these known ocean worlds. With known ocean environments it becomes important to (3) characterize their habitability and ultimately (4) search for extant life. The ROW study recommends the following high-priority targets and missions (they are all high priority and we do not prioritize between them) to address the science goals outlined in this article. The exact timing sequence of missions to execute depends on many considerations beyond the scope of ROW.

Europa: Habitability mission. Europa *Clipper* is in progress, and a Lander pre-AO study is in progress; both will/would address habitability.
Titan: Habitability and/or ocean characterization mission. *Dragonfly* is currently in the running as the next New Frontiers mission, and it would address Titan habitability (and ocean characterization to a certain extent); a Titan orbiter would address the characterization of the ocean, in particular the ice–ocean interface.
Enceladus: Search-for-life mission. A plume flyby mission with *in situ* analysis and/or sample return would address this mission goal.
Triton: Confirm-and-characterize-ocean mission. A Triton orbiter or Neptune orbiter with multiple Triton flybys (with magnetometer, gravity, thermal imagery, high-resolution imagery) would address the goals of a Triton ocean mission.

To move forward on the path toward making these missions (in addition to currently lower priority missions) a reality, ROW recommends that the following mission studies be undertaken and considered by the next Decadal Survey panel:

- Triton ocean confirmation and characterization
- Enceladus (search-for-life) and Titan (habitability and/or ocean characterization) missions or joint mission (regardless of New Frontiers 4 outcome)
- Ceres and/or Callisto missions to detect/characterize subsurface oceans/reservoirs (a Ceres study has recently been started)
- Pluto ocean characterization mission
- Ariel (and/or Miranda) confirm/characterize ocean mission (such as a Uranus flagship mission with many Ariel/Miranda flybys)

Triton, Pluto, Ceres, and Saturn system mission studies have also been recommended by the Committee on Planetary Science (CAPS) (NAS, 2017).

## Abbreviations Used

AbSciCon: Astrobiology Science Conference
CAPS: Committee on Planetary Science
CJS: Commerce, Justice, Science
COLDTech: Concepts for Ocean worlds Life Detection Technology
HW: Habitable Worlds
IR: infrared
JSO: Jupiter System Observer
KBO: Kuiper Belt object
LPSC: Lunar and Planetary Science Conference
NAI: NASA's Habitable Worlds, Exobiology, Astrobiology Institute
NOAA: National Oceanic and Atmospheric Administration
NSF: National Science Foundation
ONR: Office of Naval Research
OPAG: Outer Planets Assessment Group
PSD: Planetary Science Division
R&A: Research and Analysis
ROW: Roadmaps to Ocean Worlds
SBAG: Small Bodies Assessment Group
SLS: Space Launch System
SSW: Solar System Workings
UV: ultraviolet
